# Growth Hormone Reduces Liver Tumor Development in a MASLD-Related HCC Murine Model

**DOI:** 10.3390/ijms27219985

**Published:** 2026-11-07

**Authors:** Vanessa I. Oyarzún, Jessica Liberona, Loreto Aguilar, Nancy Solis, Paula Rivera Aguayo, Wilfredo A. González-Arriagada, Michelle S. Fernández-Cuya, Gonzalo Carrasco-Avino, Roberto Candia, Marco Arrese, Luis Antonio Díaz, Juan Pablo Arab, Claudio Cabello-Verrugio, Elisa Balboa, Lisbell D. Estrada, Jesús Llanquinao-Sandoval, Gabriel Mezzano, Francisco Barrera, Daniel Cabrera

**Affiliations:** 1 Centro de Investigación e Innovación Biomédica, Facultad de Medicina, Universidad de los Andes, Santiago 7620001, Chile; oyarzun.va@gmail.com (V.I.O.); jliberona@clinicauandes.cl (J.L.); lfaguilar@uandes.cl (L.A.); wgonzalez@uandes.cl (W.A.G.-A.); msfernandez@uandes.cl (M.S.F.-C.); 2 División de Nutrición y Diabetes, Departamento de Medicina, Clínica Universidad de los Andes, Santiago 7620157, Chile; 3 Departamento de Gastroenterología, Escuela de Medicina, Pontificia Universidad Católica de Chile, Santiago 8331150, Chile; nsolisl@gmail.com (N.S.); pxrivera@uc.cl (P.R.A.); rcandiab@ucchristus.cl (R.C.); marrese@uc.cl (M.A.); luisdiazpiga@gmail.com (L.A.D.); jparab@gmail.com (J.P.A.); 4 Facultad de Odontología, Universidad de los Andes, Santiago 7620157, Chile; 5 IMPACT Center of Interventional Medicine for Precision and Advanced Cellular Therapy, Universidad de los Andes, Santiago 7620001, Chile; 6 Anatomía Patológica, Clínica Alemana de Santiago—Facultad de Medicina, Universidad del Desarrollo, Santiago 7650568, Chile; gcarrasa@me.com; 7 Red de Salud UC CHRISTUS, Santiago 8330033, Chile; 8 MASLD Research Center, Division of Gastroenterology and Hepatology, University of California San Diego, 9452 Medical Center Dr., La Jolla, CA 92037, USA; 9 Division of Gastroenterology, Hepatology, and Nutrition, Department of Internal Medicine, Virginia Commonwealth University, Richmond, VA 23298, USA; 10 Dirección de Investigación, Universidad de Talca, Talca 3460000, Chile; claudio.cabello.verrugio@gmail.com; 11 Laboratory of Biomedical Research-LIBMED, Instituto de Investigación, Desarrollo e Innovación de Salud, Facultad de Medicina y Salud, Universidad Finis Terrae, Santiago 7501015, Chile; ebalboa@uft.cl; 12 Vicerrectoría de Vinculación con el Medio, Escuela de Kinesiología, Universidad Bernardo O’Higgins, Santiago 8370993, Chile; lisbell.estrada@ubo.cl; 13 Fundación Arturo López Pérez OECI Cancer Center, Laboratorio de Medicina Traslacional, Centro de Investigación e Innovación en Cáncer, Santiago 7500966, Chile; jesus.llanquinao@falp.org; 14 Centro de Enfermedades Digestivas, Clínica Universidad de los Andes, Santiago 7620157, Chile; gmezzano@clinicauandes.cl

**Keywords:** carcinoma, hepatocellular, metabolic dysfunction-associated steatotic liver disease, growth hormone, insulin-like growth factor 1, liver cirrhosis

## Abstract

Metabolic dysfunction-associated steatotic liver disease (MASLD)-related hepatocarcinogenesis involves metabolic injury, fibrosis, and dysregulated regenerative signaling. Because growth hormone (GH) and insulin-like growth factor 1 (IGF-1) can support repair but also activate proliferative pathways, their effects in advanced disease remain uncertain. We investigated that effect in a MASLD-related HCC model. Male C57BL/6J mice received a Western Diet (WD) and weekly carbon tetrachloride (CCl_4_) injections for 24 weeks. At week 16, mice were allocated to untreated or hormone-treated groups, with continuous subcutaneous infusion of IGF-1 at 20 or 60 µg/kg/day or GH at 0.5 or 2 mg/kg/day for 8 weeks. Both hormones reduced serum aminotransferases, lobular inflammation, hepatic collagen, and altered selected fibrosis-related transcripts, whereas IGF-1 20 µg/kg/day and GH 2 mg/kg/day reduced steatosis, and IGF-1 20 µg/kg/day and GH 0.5 mg/kg/day reduced hepatocyte ballooning. GH at 2 mg/kg/day was associated with fewer macroscopically detected hepatic surface nodules, while IGF-1 did not alter nodule burden. The GH 2 mg/kg/day group also showed a lower hepatic p-ERK/ERK ratio. Thus, continuous GH administration during late-stage WD/CCl_4_-induced hepatocarcinogenesis was associated with lower hepatic nodule burden. Histopathological confirmation of the lesions and validation in additional models are required before interpreting this finding as an antitumor or therapeutic effect.

## 1. Introduction

Hepatocellular carcinoma (HCC) is the most common primary malignancy of the liver and remains one of the leading causes of cancer-related mortality globally, projected to account for more than one million deaths annually by 2030 [1]. Importantly, although most HCC cases arise in cirrhotic livers, approximately 20–30% of MASLD-related HCC occurs in non-cirrhotic individuals [2,3]. This feature may contribute to delayed diagnosis, larger tumors at presentation, and reduced eligibility for curative therapies compared with HCC arising from other etiologies [4].

Despite recent therapeutic advances in the field, major unmet needs remain in advanced MASLD and MASLD-related HCC [5,6,7,8,9]. Resmetirom and semaglutide have expanded pharmacological options for selected patients with non-cirrhotic MASH and moderate-to-advanced fibrosis; however, these therapies do not directly address established hepatocarcinogenesis or the safety of manipulating regenerative endocrine pathways in advanced liver disease [10,11,12,13,14]. Therefore, new strategies that can modify liver injury, fibrotic remodeling, and tumor development in MASLD-related HCC are urgently needed.

One biologically plausible but controversial therapeutic target is the growth hormone-insulin-like growth factor-1 axis, hereafter referred to as the somatotropic axis [15]. This endocrine system regulates metabolism, body composition, hepatic repair, and liver regeneration [16]. In MASLD and advanced liver disease, somatotropic axis dysfunction (SAD) is common and has been associated with impaired liver function [17,18,19,20,21,22,23]. Preclinical and clinical studies suggest that modulating GH or IGF-1 signaling may ameliorate liver injury and improve metabolic homeostasis [24,25,26,27,28,29,30,31]. Both hormones are key components of the somatotropic axis. GH directly affects hepatocytes via the GH receptor (GHR), primarily through JAK2/STAT5 signaling and stimulates endogenous IGF-1 production [32,33]. The liver is the primary source of circulating IGF-1, which mediates many of GH’s systemic growth-promoting actions. However, GH also exerts direct metabolic effects on the liver that are not necessarily mediated by IGF-1 [34]. In turn, IGF-1 mediates its effects through IGF-1 receptors (IGF-1R), activating pathways such as PI3K/AKT and MAPK that regulate metabolism, cell survival, and tissue homeostasis [32,35]. Although GH and IGF-1 are physiologically linked, their hepatic effects can differ, reflecting both distinct and shared signaling mechanisms.

The relationship between the somatotropic axis and hepatocarcinogenesis is complex and highly context-dependent. GH directly stimulates GHR-dependent pathways that promote cell survival and growth. Accumulating evidence implicates GH/GHR signaling in HCC development: autocrine GH production drives proliferation, survival, migration, and invasion in HCC cells, and higher circulating GH levels together with active GHR signaling have been associated with more aggressive disease and poorer patient outcomes [36,37]. IGF-1/IGF-1R activation converges on the PI3K/AKT–mTOR and RAF/MEK/ERK pathway, and the resulting pro-survival and proliferative signal provides a plausible mechanism by which dysregulated IGF signaling could facilitate tumor progression [38,39]. Systemic somatotropic status, however, does not necessarily track with intratumoral signaling status. Hepatic GHR, STAT5, and IGF-1 expression are reduced in HCC and this reduction correlates with more aggressive tumors and poorer outcomes [40], while circulating IGF-1 levels are typically low in patients with cirrhosis and HCC, even as tumor-associated IGF signaling remains active—sustained by increased IGF-1R expression and altered IGF-2 signaling [41,42,43]. This dissociation between systemic and intratumoral IGF signaling is the main argument for characterizing local hepatic expression of somatotropic axis components directly, rather than inferring pathway activity from circulating hormone levels or systemic injury markers, when assessing the biological consequences of GH or IGF-1 supplementation in this setting. Collectively, these findings indicate that the biological output of somatotropic signaling is shaped by disease stage, hormone exposure, receptor expression, cellular context, and the relative dominance of systemic endocrine versus local autocrine/paracrine signaling.

These mechanistic links translate into genuine oncologic concerns. Chronic GH/IGF-1 excess, as in acromegaly, is associated with increased risk of several malignancies [44]. In addition, components of the IGF system—including IGF-1, IGF-2, IGF-1R, and insulin-like growth factor-binding proteins—have been implicated in tumor initiation and progression across different cancer types [45,46,47,48,49]. GH administration itself has been linked to increased cancer risk [50]. These apparent contradictions highlight a distinction that is easy to lose: chronic hormone excess, physiological replacement of a deficient axis, and local tumor-autonomous activation of GH/IGF-1 signaling are not biologically equivalent, and each carries different oncologic implications [51,52]. It also justifies rigorously testing the safety of somatotropic axis-based interventions specifically in the setting of advanced fibrosis, cirrhosis, and HCC, rather than extrapolating from other clinical populations.

Here we administered GH and IGF-1 separately to isolate the direct effects of each hormone using two doses of each hormone selected from the literature and our previous preclinical work, and evaluated differences between the two doses under advanced hepatic disease (see Materials and Methods). We had previously characterized somatotropic axis dysfunction in a diet-induced, non-tumoral model of early-stage MASLD, where hepatic steatosis and metabolic alterations coincided with reduced circulating IGF-1 [24], and shown that GH or IGF-1 supplementation in that model improved aminotransferases, hepatic steatosis, and associated systemic alterations [25]. The present study asks whether this benefit persists in a fundamentally different disease context—once MASLD has progressed to advanced fibrosis and HCC—and, critically, whether modulating the somatotropic axis at this stage affects tumor development itself. Whether altering GH or IGF-1 signaling is harmful, neutral, or protective in MASLD-related HCC remains unresolved. We hypothesized that, in the setting of established advanced liver disease, somatotropic intervention could shift the balance among hepatocellular injury, fibrogenic remodeling, tissue repair, and pro-tumorigenic signaling in either direction. We therefore tested the effect of late-phase GH or IGF-1 administration on tumor development in a mouse model of MASLD-related cirrhosis and HCC, evaluating hepatic surface nodule incidence, number, and size alongside molecular markers of liver injury, fibrosis, somatotropic signaling, and proliferative pathway activation.

## 2. Results

### 2.1. Endpoint Anthropometric and Hepatic Features Following Late-Phase IGF-1 or GH Supplementation

**Figure 1:**
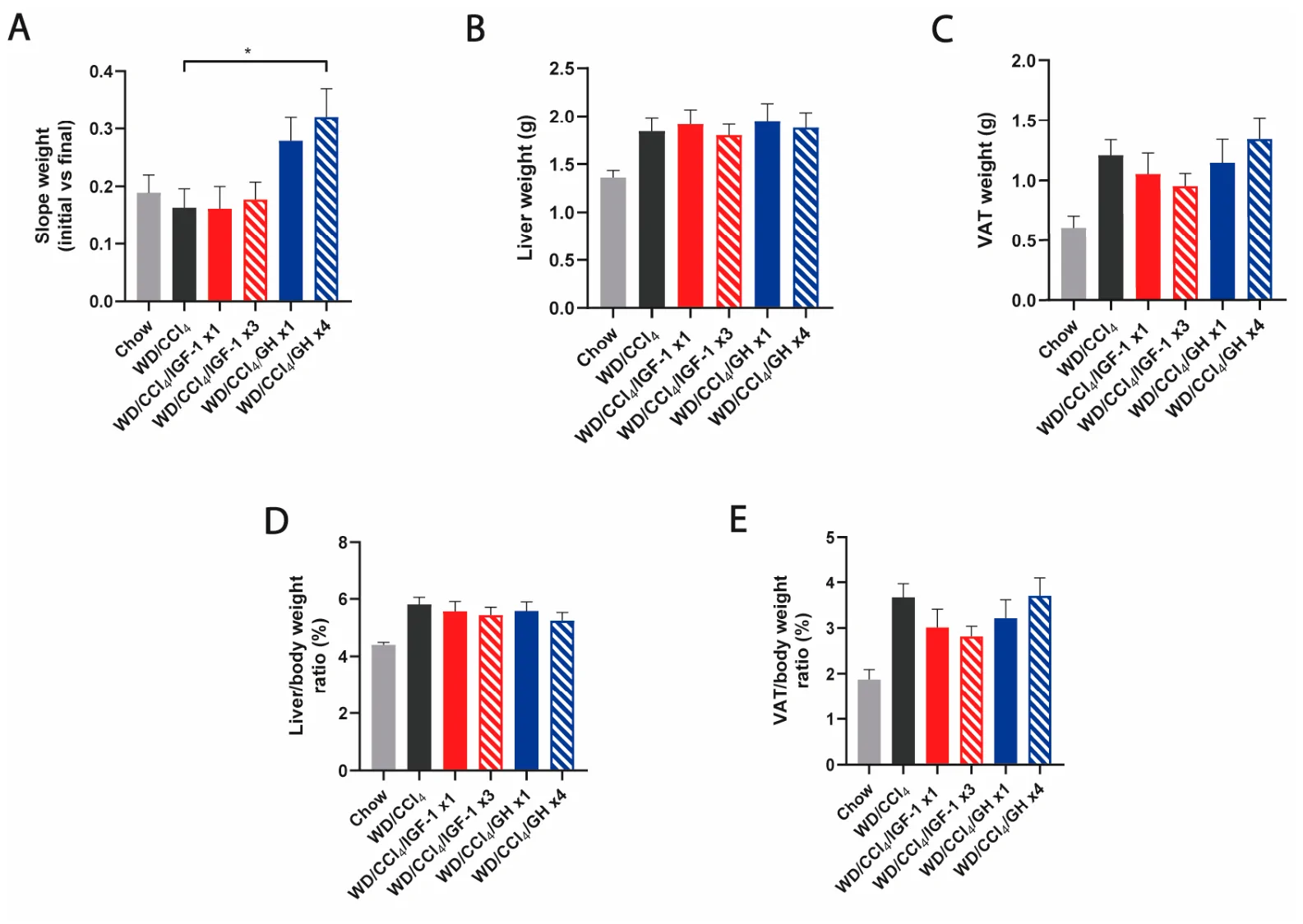
Effects of IGF-1 and GH on anthropometric parameters in late-stage MASLD-related HCC model. (**A**) Slope graph showing body weight at the beginning and at the end of the experimental protocol (* *p* < 0.05, one-way ANOVA). (**B**) Liver weight (g) (not significant, one-way ANOVA). (**C**) Liver-to-body weight ratio (%) (not significant, Kruskal–Wallis). (**D**) Visceral adipose tissue (VAT) weight (g) (not significant, Kruskal–Wallis). (**E**) VAT-to-body ratio (%) (not significant, Kruskal–Wallis). (Chow, *n* = 12; WD/CCl_4_, *n* = 12; WD/CCl_4_/IGF-1 x1, *n* = 8; WD/CCl_4_/IGF-1 x3, *n* = 10; WD/CCl_4_/GH x1, *n* = 10; WD/CCl_4_/GH x4, *n* = 9). IGF-1 x1: 20 µg/kg/day; IGF- 1 x3: 60 µg/kg/day; GH x1: 0.5 mg/kg/day; GH x4: 2 mg/kg/day. Data are presented as mean ± SEM.

Anthropometric parameters and liver pathology were assessed at the 24-week endpoint in chow-fed controls and in mice exposed to WD/CCl_4_ with or without hormone supplementation (Supplementary Figure S1 and Figure 1A–E). At this endpoint, untreated WD/CCl_4_ mice displayed steatosis, hepatocellular ballooning, inflammatory infiltrates, extensive collagen deposition, bridging fibrosis (F3), and macroscopically visible hepatic surface nodules. These observations document an advanced liver disease phenotype in the untreated WD/CCl_4_ group at week 24. Because no independent cohort was evaluated at week 16, these endpoint findings do not establish the presence or severity of HCC before hormone administration.

We next examined whether supplementation with IGF-1 or GH during weeks 16–24 was associated with differences in anthropometric parameters at the study endpoint. Mice receiving GH at 2 mg/kg/day had a higher final body weight than untreated WD/CCl_4_ mice (35.5 g vs. 32.3 g; *p* = 0.0395; Figure 1A). In contrast, liver weight, visceral adipose tissue mass, liver-to-body weight ratio, and visceral adipose tissue-to-body weight ratio did not differ significantly between the untreated WD/CCl_4_ group and any of the IGF-1- or GH-treated groups (Figure 1B–E). Thus, late-phase hormone supplementation had limited effects on the anthropometric endpoints evaluated, except for the higher final body weight observed in mice receiving GH at 2 mg/kg/day. This difference was not accompanied by an increase in liver weight or visceral adipose tissue mass.

### 2.2. IGF-1 and GH Reduce Serum Aminotransferases and Improve Liver Histology but Have Heterogeneous Effects on Lipogenic Outcomes

**Figure 2:**
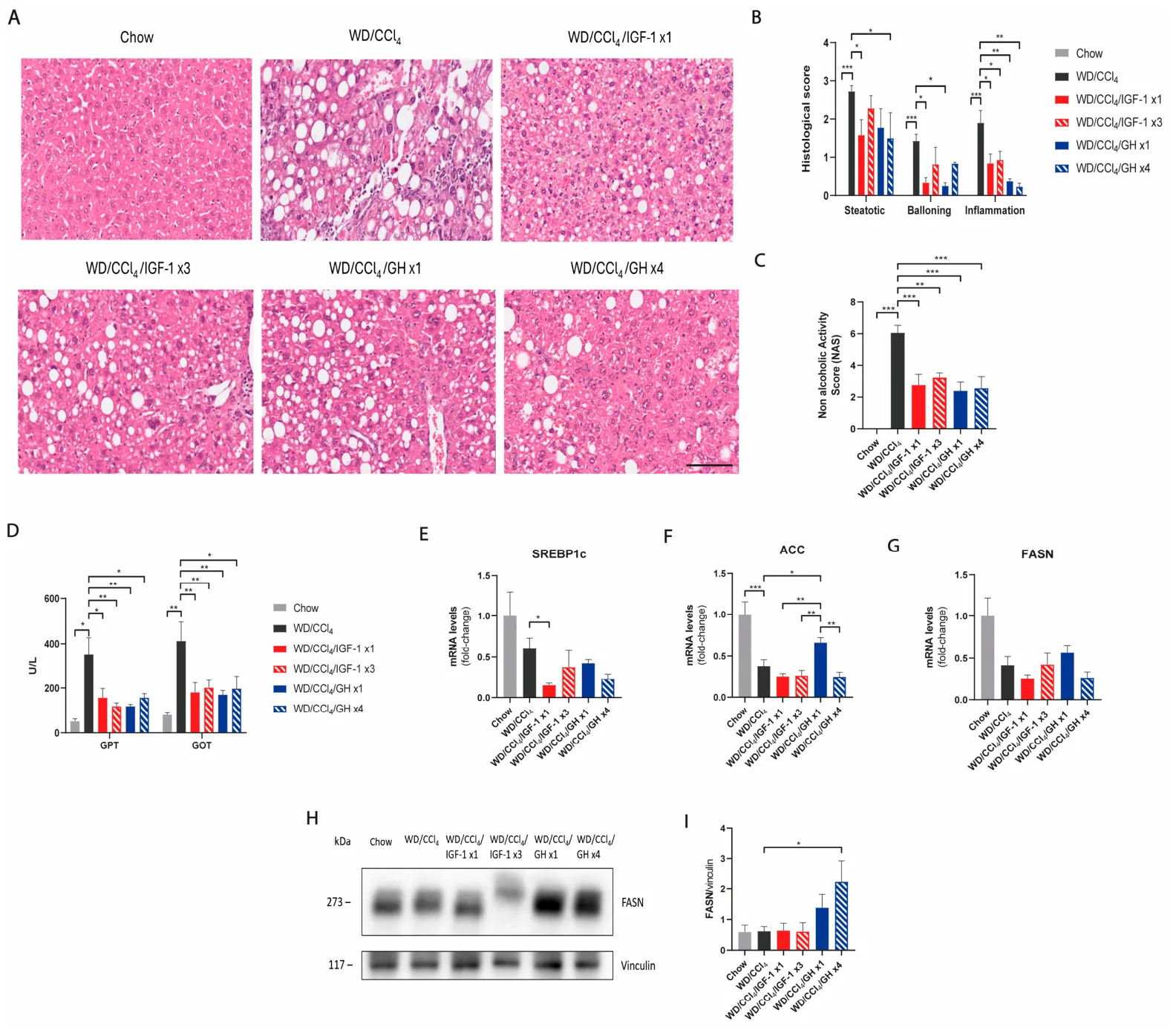
Effects of IGF-1 and GH supplementation on liver histology, serum transaminases, and hepatic lipogenic gene expression in MASLD-related HCC model. (**A**) Representative hematoxylin and eosin (H&E)-stained liver sections from mice fed a chow diet (Chow), a Western diet plus CCl_4_ treatment (WD/CCl_4_), or WD/CCl_4_ with two doses of IGF-1 (IGF-1 x1: 20 µg/kg/day and IGF-1 x3: 60 µg/kg/day) or GH (GH x1: 0.5 mg/kg/day and GH x4: 2 mg/kg/day). Scale bar: 50 µm. (**B**) Histological score (Chow, *n* = 4; WD/CCl_4_, *n* = 4; WD/CCl_4_/IGF-1 x1, *n* = 5; WD/CCl_4_/IGF-1 x3, *n* = 5; WD/CCl_4_/GH x1, *n* = 4; WD/CCl_4_/GH x4, *n* = 3; * *p* < 0.05; ** *p* < 0.01; *** *p* < 0.001, two-way ANOVA). (**C**) Nonalcoholic fatty liver disease activity score (NAS) (Chow, *n* = 4; WD/CCl_4_, *n* = 4; WD/CCl_4_/IGF-1 x1, *n* = 5; WD/CCl_4_/IGF-1 x3, *n* = 5; WD/CCl_4_/GH x1, *n* = 4; WD/CCl_4_/GH x4, *n* = 3; ** *p* < 0.01; *** *p* < 0.001, one-way ANOVA). (**D**) Serum levels of glutamate pyruvate transaminase (GPT/ALT) and glutamate-oxaloacetate transaminase (GOT/AST) (Chow, *n* = 3; WD/CCl_4_, *n* = 11; WD/CCl_4_/IGF-1 x1, *n* = 8; WD/CCl_4_/IGF-1 x3, *n* = 10; WD/CCl_4_/GH x1, *n* = 10; WD/CCl_4_/GH x4, *n* = 8; * *p* < 0.05; ** *p* < 0.01, mixed-effects models). (**E**–**G**) Hepatic mRNA expression levels of sterol regulatory element-binding protein 1 (SREBP-1c), ccetyl-CoA carboxylase (ACC), and fatty acid synthase (FASN) (Chow, *n* = 5; WD/CCl_4_, *n* = 8; WD/CCl_4_/IGF-1 x1, *n* = 6; WD/CCl_4_/IGF-1 x3, *n* = 6; WD/CCl_4_/GH x1, *n* = 8; WD/CCl_4_/GH x4, *n* = 7; * *p* < 0.05; ** *p* < 0.01; *** *p* < 0.001, one-way ANOVA or Kruskal–Wallis). (**H**) Western blot analyses of FASN protein in hepatic tissue. Vinculin was used as a loading control. Numbers on the left side of the panel indicate molecular weight (kDa). (**I**) Densitometric quantification of FASN protein levels normalized to he loading control, vinculin (Chow, *n* = 7; WD/CCl_4_, *n* = 6; WD/CCl_4_/IGF-1 x1, *n* = 6; WD/CCl_4_/IGF-1 x3, *n* = 4; WD/CCl_4_/GH x1, *n* = 8; WD/CCl_4_/GH x4, *n* = 6; * *p* < 0.05, one-way ANOVA). Data are presented as mean ± SEM.

We next assessed liver histology and serum aminotransferase levels at the 24-week endpoint in untreated WD/CCl_4_ mice and in mice receiving IGF-1 or GH during weeks 16–24 (Figure 2A–C). Both IGF-1 and GH significantly reduced lobular inflammation score compared with the untreated WD/CCl_4_ group (Figure 2A,B). In addition, hepatocyte ballooning scores were lower in mice receiving IGF-1 at 20 µg/kg/day and GH at 0.5 mg/kg/day than in untreated WD/CCl_4_ mice (*p* = 0.0274 and *p* = 0.0224, *respectively*; Figure 2A,B). Additionally, steatosis score was significantly lower with IGF-1 at 20 µg/kg/day and GH at 2 mg/kg/day compared to untreated WD/CCl_4_ group (Figure 2A,B). All IGF-1- and GH-treated groups had a significantly lower NAS score compared to untreated WD/CCl_4_ group (Figure 2C).

Consistent with the histological improvement, all IGF-1- and GH-treated groups had lower serum aminotransferase levels than the untreated WD/CCl_4_ group. Serum GPT/ALT levels were 350.8 ± 75.6 U/L in untreated WD/CCl_4_ mice, compared with 157.1 ± 41.4 U/L in the IGF-1 20 µg/kg/day group (*p* = 0.0279) and 119.2 ± 14.8 U/L in the IGF-1 60 µg/kg/day group (*p* = 0.0021). Serum GOT/AST levels were also lower in both IGF-1 groups: 180.7 ± 43.8 and 201.6 ± 33.2 U/L, respectively, compared with 412.0 ± 83.2 U/L in untreated WD/CCl_4_ mice (*p* = 0.0081 and *p* = 0.0079, *respectively*; Figure 2D).

Similarly, serum GPT/ALT levels were lower in mice receiving GH at 0.5 mg/kg/day (119.4 ± 7.6 U/L; *p* = 0.0029) or 2 mg/kg/day (157.1 ± 17.9 U/L; *p* = 0.028) than in untreated WD/CCl_4_ mice. Serum GOT/AST levels were 170.3 ± 19.0 U/L in the GH 0.5 mg/kg/day group (*p* = 0.0017) and 197.0 ± 56.6 U/L in the GH 2 mg/kg/day group (*p* = 0.0116), compared with 412.0 ± 83.2 U/L in the untreated WD/CCl_4_ group (Figure 2D).

These findings demonstrate that IGF-1 and GH administration reduced circulating aminotransferase levels and improved liver histology, with lower lobular inflammation across all treatment groups and reduction in steatosis and hepatocyte ballooning in selected groups, accompanied by lower NAS scores.

To further characterize the hepatic response to IGF-1 and GH administration, we measured selected lipogenesis-related markers at the 24-week endpoint, including sterol regulatory element-binding protein 1c (SREBP-1c), acetyl-CoA carboxylase (ACC), and fatty acid synthase (FASN) (Figure 2E–I). SREBP-1c mRNA expression in mice receiving IGF-1 at 20 µg/kg/day was 0.25-fold that of the untreated WD/CCl_4_ group (*p* = 0.0268; Figure 2E). No significant difference was detected in the IGF-1 60 µg/kg/day or either GH group.

ACC mRNA expression in mice receiving GH at 0.5 mg/kg/day was 1.75-fold that of untreated WD/CCl_4_ mice (*p* = 0.0254). ACC expression in this group was also 2.6-fold that of the IGF-1 20 µg/kg/day group, 2.5-fold that of the IGF-1 60 µg/kg/day group, and 2.6-fold that of the GH 2 mg/kg/day group (*p* < 0.01 for all comparisons; Figure 2F). No treatment group differed significantly from the untreated WD/CCl_4_ group in FASN mRNA expression (Figure 2G).

At the protein level, hepatic FASN abundance in mice receiving GH at 2 mg/kg/day was 3.6-fold that of untreated WD/CCl_4_ mice (*p* = 0.036; Figure 2H,I). FASN protein abundance did not differ significantly between the untreated WD/CCl_4_ group and mice receiving either dose of IGF-1 or GH at 0.5 mg/kg/day.

Overall, the changes in lipogenesis-related markers were heterogeneous and were not coordinated across transcripts and protein. IGF-1 at 20 µg/kg/day was associated with lower SREBP-1c mRNA, GH at 0.5 mg/kg/day with higher ACC mRNA, and GH at 2 mg/kg/day with higher FASN protein abundance. Despite the increase in FASN protein abundance, GH at 2 mg/kg/day was associated with reduced hepatic steatosis. Therefore, the present data indicate selective changes in lipogenesis-related markers but does not demonstrate a consistent increase or decrease in hepatic de novo lipogenesis.

At the 24-week endpoint, untreated WD/CCl_4_ mice showed lower hepatic expression of the evaluated lipogenesis-related genes than WD-only mice (overall group effect, *p* < 0.01; Supplementary Figure S2). This endpoint comparison demonstrates lower expression in the WD/CCl_4_ group but does not establish when this difference emerged or whether it reflects reduced metabolic plasticity. Overall, IGF-1 and GH administration during weeks 16–24 was associated with lower serum aminotransferase levels and improved liver histology, as reflected by lower NAS scores, whereas changes in lipogenesis-related markers were heterogeneous and were not coordinated across transcripts and protein.

### 2.3. IGF-1 and GH Reduce Hepatic Collagen and Selected Fibrosis-Related Transcripts but Have Heterogeneous Effects on Inflammatory Markers

**Figure 3:**
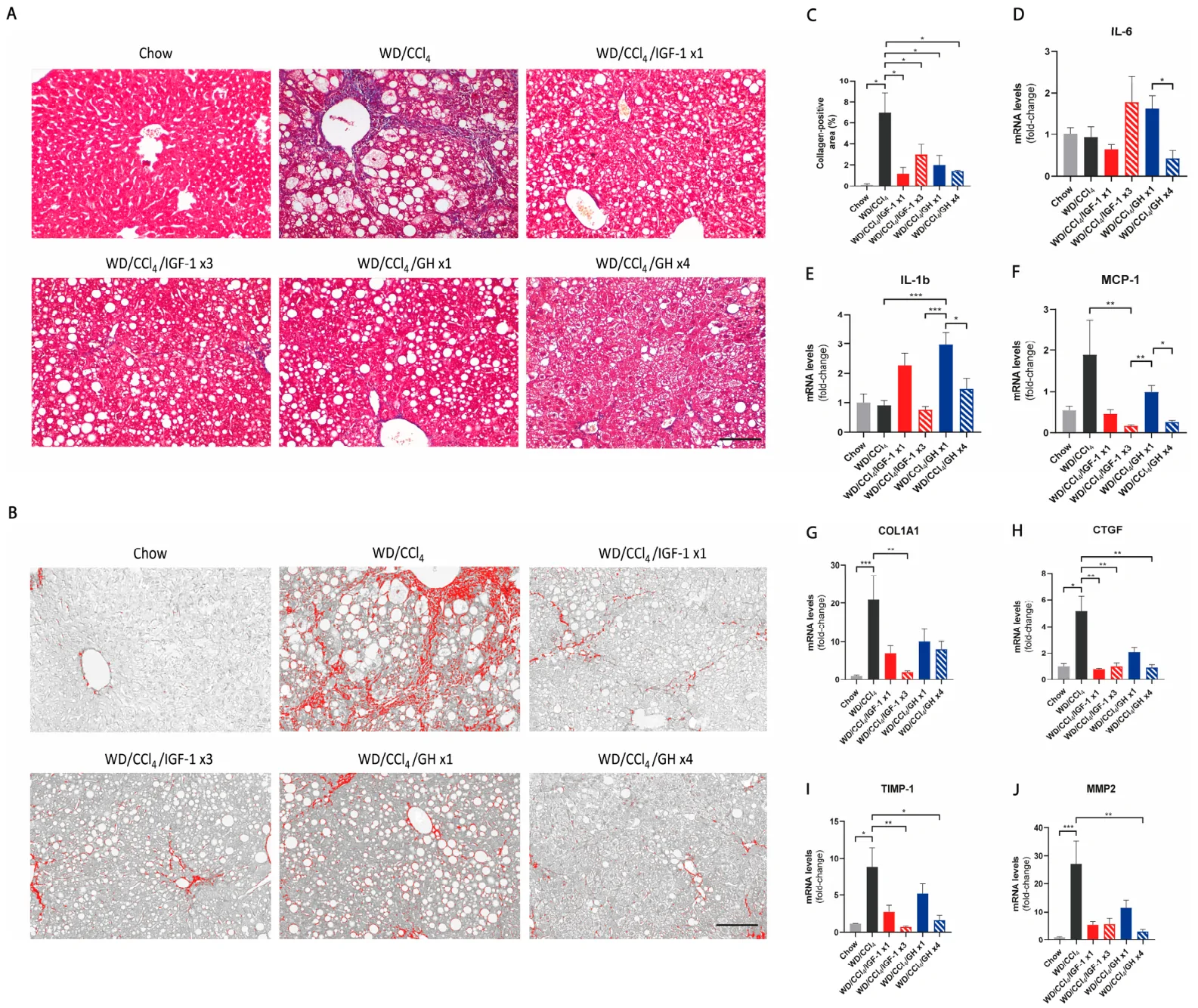
GH and IGF-1 supplementation are associated with lower hepatic collagen and reduced selected fibrosis-related markers in MASLD-related HCC model. (**A**) Representative Masson’s trichrome-stained liver sections from mice fed a chow diet (Chow), a western diet plus CCl_4_ treatment (WD/CCl_4_), or WD/CCl_4_ with two doses of IGF-1 (IGF-1 x1: 20 µg/kg/day and IGF-1 x3: 60 µg/kg/day) or GH (GH x1: 0.5 mg/kg/day and GH x4: 2 mg/kg/day). Collagen fibers stain blue, cytoplasm and muscle fibers stain red, and nuclei appear dark. Scale bar: 50 µm. (**B**) Representative images of collagen-positive area segmentation using ImageJ software. Scale bar: 50 µm. (**C**) Quantification of collagen-positive area (%) in Masson’s trichrome-stained liver sections. (Chow, *n* = 2; WD/CCl_4_, *n* = 3; WD/CCl_4_/IGF-1 x1, *n* = 3; WD/CCl_4_/IGF-1 x3, *n* = 3; WD/CCl_4_/GH x1, *n* = 3; WD/CCl_4_/GH x4, *n* = 2; * *p* < 0.05, one-way ANOVA). (**D**–**F**) Hepatic mRNA expression levels of interleukin 6 (IL-6), interleukin 1 beta (IL-1β), and monocyte chemoattractant protein-1 (MCP-1), which are inflammation-related genes (Chow, *n* = 5; WD/CCl_4_, *n* = 8; WD/CCl_4_/IGF-1 x1, *n* = 6; WD/CCl_4_/IGF-1 x3, *n* = 6; WD/CCl_4_/GH x1, *n* = 8; WD/CCl_4_/GH x4, *n* = 7; * *p* < 0.05; ** *p* < 0.01; *** *p* < 0.001, one-way ANOVA or Kruskal–Wallis). (**G**–**J**) Hepatic mRNA levels of α-1 type I collagen (COL1A1), connective tissue growth factor (CTGF), tissue inhibitor of metalloproteinase-1 (TIMP-1), and matrix metalloproteinase 2 (MMP2), which are fibrosis-related genes (Chow, *n* = 5; WD/CCl_4_, *n* = 8; WD/CCl_4_/IGF-1 x1, *n* = 6; WD/CCl_4_/IGF-1 x3, *n* = 6; WD/CCl_4_/GH x1, *n* = 8; WD/CCl_4_/GH x4, *n* = 7; * *p* < 0.05; ** *p* < 0.01; *** *p* < 0.001, one-way ANOVA or Kruskal–Wallis). Data are presented as mean ± SEM.

We next evaluated fibrosis- and inflammation-related endpoints at the 24-week study endpoint, including collagen staining, collagen type I protein abundance, and hepatic expression of selected genes (Supplementary Figure S3 and Figure 3A–J). Representative Masson’s trichrome-stained sections from untreated WD/CCl_4_ mice showed extensive collagen deposition and bridging fibrosis (F3). Sections from IGF-1- and GH-treated mice appeared to show less collagen staining, predominantly in portal and periportal regions (F1c and F2) (Figure 3A). Quantified collagen staining showed a significant increase to hepatic collagen-positive area in the untreated WD/CCl_4_ mice compared to chow mice (*p* = 0.0124; Figure 3B,C). In mice, IGF-1 at 20 µg/kg/day and IGF-1 at 60 µg/kg/day significantly reduce collagen compared with untreated WD/CCl_4_ mice (*p* = 0.0142 and *p* = 0.0277, *respectively*; Figure 3B,C). Similarly, GH at 0.5 mg/kg/day and GH at 2 mg/kg/day significantly reduce collagen compared with untreated WD/CCl_4_ mice (*p* = 0.0255 for both comparisons; Figure 3B,C). However, these histological differences were not accompanied by significant changes in collagen type I protein abundance, which did not differ significantly between untreated WD/CCl_4_ mice and any of the IGF-1- or GH-treated groups (Supplementary Figure S3).

Hepatic expression of selected fibrosis-related genes differed according to hormone and dose. In mice receiving IGF-1 at 60 µg/kg/day, COL1A1, CTGF, and TIMP-1 mRNA levels were 0.095-, 0.20-, and 0.08-fold those of untreated WD/CCl_4_ mice, respectively (*p* = 0.0099, *p* = 0.0061, and *p* = 0.0093, *respectively*, for comparisons; Figure 3G–I). In mice receiving GH at 2 mg/kg/day, CTGF, TIMP-1, and MMP2 mRNA levels were 0.18-, 0.18-, and 0.11-fold those of untreated WD/CCl_4_ mice, respectively (*p* = 0.0048, *p* = 0.0183, and *p* = 0.0058, *respectively*; Figure 3H–J). CTGF mRNA expression was also lower in mice receiving IGF-1 at 20 µg/kg/day, reaching 0.15-fold that of untreated WD/CCl_4_ mice (*p* = 0.0019; Figure 3H). No other significant differences in the evaluated fibrosis-related transcripts were detected in the IGF-1 20 µg/kg/day or GH 0.5 mg/kg/day groups.

We also evaluated hepatic expression of selected inflammation-related genes. MCP-1 mRNA expression in mice receiving IGF-1 at 60 µg/kg/day was 0.085-fold that of untreated WD/CCl_4_ mice (*p* = 0.0037), whereas the IGF-1 20 µg/kg/day group did not differ significantly from untreated WD/CCl_4_ mice (Figure 3D–F). In contrast, IL-1β mRNA expression in mice receiving GH at 0.5 mg/kg/day was 3.26-fold that of untreated WD/CCl_4_ mice (*p* < 0.001). This difference was not accompanied by significant changes in IL-6 or MCP-1 relative to the untreated WD/CCl_4_ group. None of the three inflammation-related transcripts differed significantly between mice receiving GH at 2 mg/kg/day and untreated WD/CCl_4_ mice. However, IL-6, IL-1β, and MCP-1 mRNA levels were higher in the GH 0.5 mg/kg/day group than in the GH 2 mg/kg/day group (*p* = 0.0201, *p* = 0.0202, and *p* = 0.0397, *respectively*, for comparisons; Figure 3D–F).

Overall, IGF-1 at 60 µg/kg/day and GH at 2 mg/kg/day were associated with lower expression of selected fibrosis-related transcripts. Quantitative Masson’s trichrome analysis showed a lower collagen-positive area in all IGF-1- and GH-treated mice. However, these changes were not accompanied by a reduction in collagen type I protein abundance. The effects on inflammation-related transcripts were heterogeneous, including lower MCP-1 expression with IGF-1 at 60 µg/kg/day and higher IL-1β expression with GH at 0.5 mg/kg/day. These findings indicate a reduction in hepatic collagen deposition in both IGF-1 and GH-treated mice, alongside changes in selected fibrosis-related transcripts, without a consistent anti-inflammatory effect.

### 2.4. GH at 2 mg/kg/day Is Associated with Lower Hepatic Surface Nodule Burden at the 24-Week Endpoint

**Figure 4:**
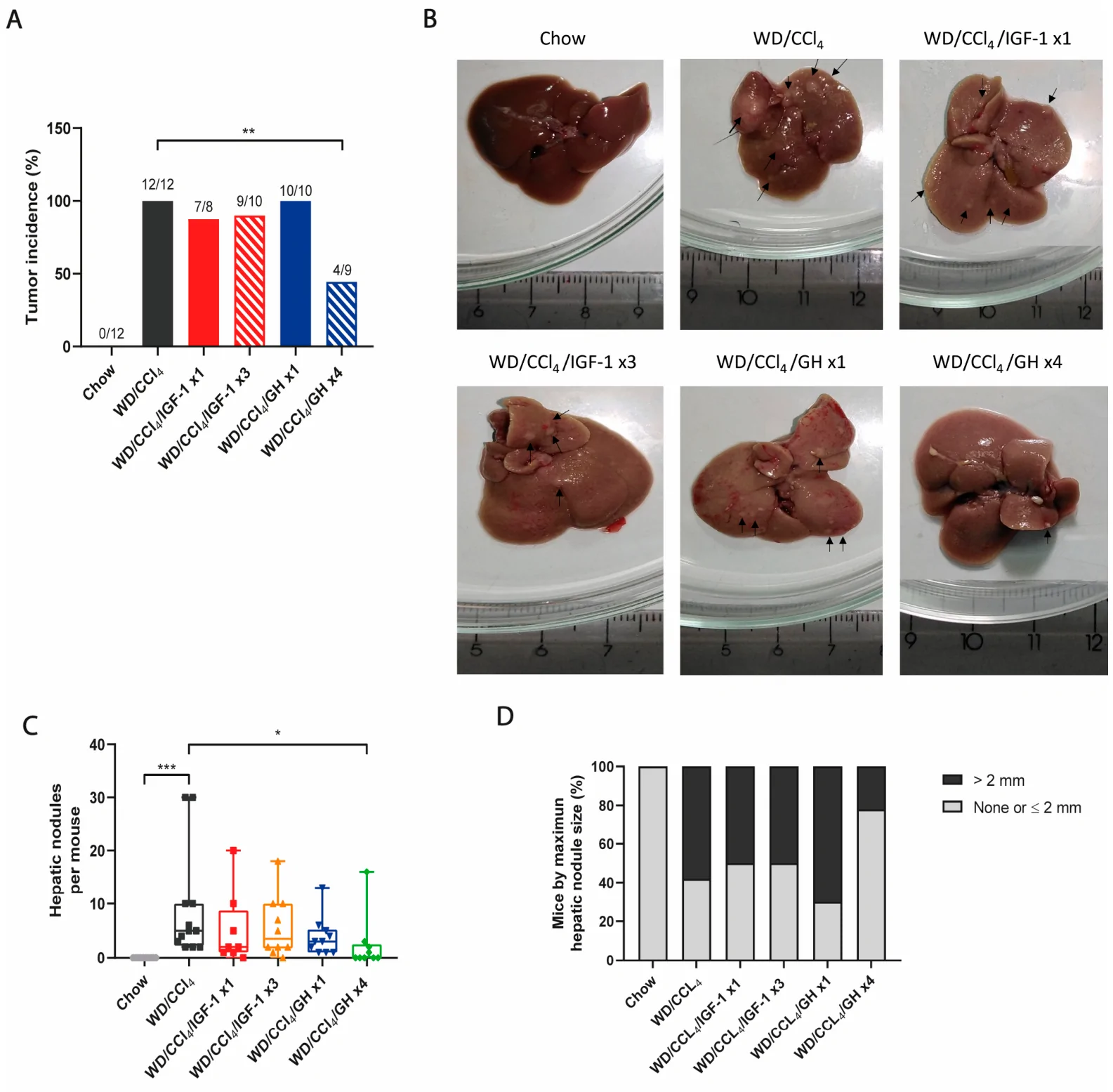
GH at 2 mg/kg/day reduced hepatic nodules in the MASLD-related HCC mouse model. (**A**) Hepatic surface nodule incidence per mouse in the study groups at 24 weeks after WD feeding, weekly CCl_4_ injections, and supplementation with IGF-1 and GH. The groups include mice on a chow diet (Chow), a western diet plus CCl_4_ treatment (WD/CCl_4_), or WD/CCl_4_ combined with two doses of IGF-1 (IGF-1 x1: 20 µg/kg/day and IGF-1 x3: 60 µg/kg/day) or GH (GH x1: 0.5 mg/kg/day and GH x4: 2 mg/kg/day; ** *p* < 0.01, Fisher’s exact test). (**B**) Representative macroscopic liver images. Black arrows indicate hepatic nodules. (**C**) Number of hepatic nodules per mouse (* *p* < 0.05; *** *p* < 0.001, Kruskal–Wallis). Data are presented as mean ± SEM. (**D**) Distribution of mice according to maximum hepatic nodule diameter. Data are presented as the percentage of mice in each category. (Chow, *n* = 12; WD/CCl_4_, *n* = 12; WD/CCl_4_/IGF-1 x1, *n* = 8; WD/CCl_4_/IGF-1 x3, *n* = 10; WD/CCl_4_/GH x1, *n* = 10; WD/CCl_4_/GH x4, *n* = 9).

At the 24-week endpoint, we evaluated the incidence, number, and size of macroscopically visible hepatic surface nodules in untreated WD/CCl_4_ mice and in mice that received IGF-1 or GH during weeks 16–24 (Figure 4A–D). Because the lesions were not histopathologically classified, they are referred to as hepatic surface nodules rather than tumors or HCC lesions.

The proportion of mice with at least one hepatic surface nodule did not differ significantly between the untreated WD/CCl_4_ group and mice receiving IGF-1 at 20 or 60 µg/kg/day or GH at 0.5 mg/kg/day. In the GH 2 mg/kg/day group, nodules were detected in 4 of 9 mice, compared with 12 of 12 untreated WD/CCl_4_ mice (Fisher’s exact test, *p* = 0.062; Figure 4A). Representative macroscopic images showed multiple hepatic surface nodules in untreated WD/CCl_4_ mice (Figure 4B). Mice receiving GH at 2 mg/kg/day also had fewer hepatic surface nodules per animal than untreated WD/CCl_4_ mice (2.3 ± 1.7 vs. 9.0 ± 2.9 nodules per mouse; *p* = 0.043; Figure 4C). The number of nodules per mouse did not differ significantly between the untreated WD/CCl_4_ group and either IGF-1 group or the GH 0.5 mg/kg/day group.

To evaluate lesion size at the animal level, mice were categorized according to the maximum diameter of the largest surface nodule as having no nodules, nodules ≤ 2 mm, or nodules > 2 mm. The distribution of these categories did not differ significantly between the GH 2 mg/kg/day and untreated WD/CCl_4_ group, although fewer GH-treated mice had a maximum nodule diameter > 2 mm (Fisher’s exact test, *p* = 0.1842) (Figure 4D). No significant differences in maximum nodule diameter were detected for either IGF-1 group or the GH 0.5 mg/kg/day group.

Overall, GH administration at 2 mg/kg/day during weeks 16–24 was associated with a lower hepatic surface nodule burden at the study endpoint. This finding was not observed with GH at 0.5 mg/kg/day or with either dose of IGF-1. Histopathological classification of the lesions is required before this result can be interpreted as evidence of a reduction in HCC burden.

### 2.5. GH at 2 mg/kg/day Is Associated with Reduced ERK Phosphorylation and a Distinct Somatotropic-Axis Transcriptional Profile

To identify molecular correlates of the lower hepatic surface nodule burden observed with GH at 2 mg/kg/day, we examined ERK phosphorylation at the 24-week endpoint. We focused on the RAF–MEK–ERK pathway because GH can regulate this pathway in some cellular contexts [53] and sustained ERK activation has been implicated in HCC progression [54,55,56].

**Figure 5:**
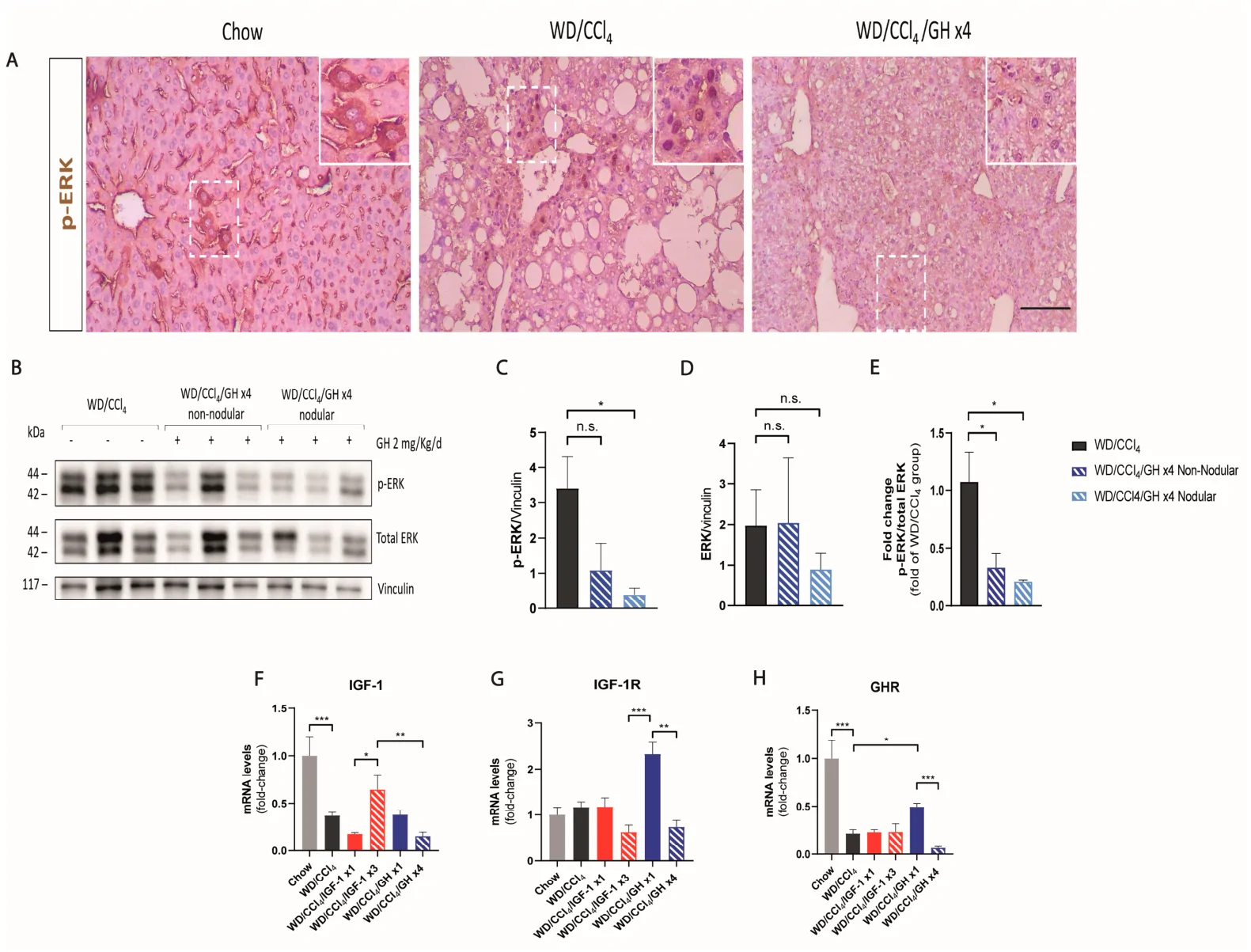
GH at 2 mg/kg/day is associated with a lower hepatic p-ERK/total ERK ratio in MASLD-related HCC. (**A**) Immunohistochemistry of p-ERK in liver tissue from mice fed a chow diet (Chow), a western diet plus CCl_4_ treatment (WD/CCl_4_), and WD/CCl_4_ treated with GH at 2 mg/kg/day (GH x4). p-ERK shows a cytoplasmic localization pattern in all groups (Insets, white arrowheads), whereas the WD/CCl_4_ group also showed a nuclear localization pattern (Insets). Scale bar: 50 µm. (**B**) Western blot analyses of p-ERK (1/2) and total ERK (1/2) expression in hepatic tissue from WD/CCl_4_ group and GH x4 groups, including non-nodular and nodular hepatic tissue for GH x4 groups. Vinculin was used as a loading control. Numbers on the left side of the panel indicate molecular weight (kDa). (**C**) Densitometric quantification of p-ERK (1/2) protein levels normalized to vinculin. (**D**) Total ERK (1/2) protein levels normalized to vinculin. (**E**) p-ERK/total ERK ratio calculated from the vinculin-normalized values. Analysis was performed using independent liver samples from untreated WD/CCl_4_ mice and nodular and non-nodular hepatic regions from GH x4-treated mice (2 mg/kg/day) (WD/CCl_4_, *n* = 3; WD/CCl_4_/GH x4 non-nodular, *n* = 3; WD/CCl_4_/GH x4 nodular, *n* = 3; * *p* < 0.05, n.s. not significant, one-way ANOVA). (**F**–**H**) Was performed to assess the hepatic mRNA level of insulin-like growth factor 1 (IGF-1), insulin-like growth factor 1 receptor (IGF-1R), and growth hormone receptor (GHR), which are somatotropic axis-related genes (Chow, *n* = 5; WD/CCl_4_, *n* = 8; WD/CCl_4_/IGF-1 x1, *n* = 6; WD/CCl_4_/IGF-1 x3, *n* = 6; WD/CCl_4_/GH x1, *n* = 8; WD/CCl_4_/GH x4, *n* = 7; * *p* < 0.05; ** *p* < 0.01; *** *p* < 0.001, one-way ANOVA or Kruskal–Wallis). Data are presented as mean ± SEM.

Representative immunohistochemical images showed predominantly cytoplasmic and relatively homogeneous p-ERK staining in chow-fed mice. In untreated WD/CCl_4_ mice, p-ERK staining was more heterogeneous and included an apparent nuclear signal. In contrast, sections from mice receiving GH at 2 mg/kg/day showed predominantly cytoplasmic staining with less evident nuclear p-ERK signal (Figure 5A).

Immunoblot analysis was consistent with immunohistochemical observations. p-ERK abundance was lower in nodular regions from mice receiving GH at 2 mg/kg/day than in untreated WD/CCl_4_ liver samples (*p* = 0.04), whereas the difference in non-nodular regions did not reach statistical significance (Figure 5B,C). No statistically significant difference in total ERK abundance were detected among the evaluated groups (Figure 5D). The p-ERK/ERK ratio was lower in both nodular and non-nodular regions from GH-treated mice than in untreated WD/CCl_4_ liver samples (*p* = 0.0355 and *p* = 0.0191, *respectively*, for both comparisons; Figure 5E). The corresponding uncropped immunoblots and detailed results are provided in Supplementary Figure S4 and Supplementary Table S1, respectively.

We next examined whether the lower nodule burden and ERK phosphorylation observed with GH at 2 mg/kg/day were accompanied by changes in hepatic expression of somatotropic-axis components. GH at 2 mg/kg/day did not significantly increase GHR, IGF-1, or IGF-1R mRNA expression relative to untreated WD/CCl_4_ mice (Figure 5F–H). In contrast, GH at 0.5 mg/kg/day increased GHR mRNA expression relative to both untreated WD/CCl_4_ mice (*p* = 0.0398) and mice receiving GH at 2 mg/kg/day (*p* < 0.001; Figure 5H). IGF-1R mRNA expression was also higher with GH at 0.5 mg/kg/day than with GH at 2 mg/kg/day (*p* = 0.019; Figure 5G). These results demonstrate a dose-specific transcriptional response rather than uniform activation of the hepatic somatotropic axis.

IGF-1 administration produced a different expression pattern. Neither dose significantly altered GHR, IGF-1, or IGF-1R mRNA expression relative to untreated WD/CCl_4_ mice. However, IGF-1 mRNA expression was higher with IGF-1 at 60 µg/kg/day than with IGF-1 at 20 µg/kg/day (*p* < 0.05; Figure 5F).

Collectively, GH at 2 mg/kg/day was associated with lower p-ERK/total ERK ratio in both nodular and non-nodular liver samples, without a concomitant increase in hepatic GHR, IGF-1, or IGF-1R mRNA expression. This molecular profile coincided with the lower hepatic surface nodule burden observed at the same dose, although these findings do not establish a causal relationship between ERK phosphorylation and nodule burden.

## 3. Discussion

The most important observation in this study was the dose-specific response to GH. Administration of GH at 2 mg/kg/day during the final eight weeks of the WD/CCl_4_ protocol was associated with a lower burden of macroscopically detected hepatic surface nodules and a lower hepatic p-ERK/ERK ratio at the 24-week endpoint. These findings were not reproduced by GH at 0.5 mg/kg/day or by either dose of IGF-1, highlighting the specificity of the response. Both hormones also reduced serum aminotransferase levels, hepatic collagen and modified selected fibrosis-related transcripts. Overall, the findings indicate that modulation of the somatotropic axis can produce distinct and potentially beneficial hepatic effects, depending on the hormone, dose, and biological outcome evaluated.

The timing of the intervention is important for interpreting these findings. In the original characterization of the WD/CCl_4_ model, advanced fibrosis develops before overt HCC is typically detected at approximately 24 weeks [57]. In the present study, hormone administration began at week 16, whereas liver histology and hepatic surface nodules were evaluated at the 24-week endpoint. Because no independent cohort was examined at week 16, the study does not establish that all animals had HCC when treatment began. The results are therefore most appropriately interpreted as effects occurring during a late fibrotic and carcinogenic phase of the model, rather than as regression of confirmed pre-existing HCC. Nevertheless, the lower nodule burden observed with GH at 2 mg/kg/day suggests that this treatment may have influenced lesion development or progression during this advanced stage of disease.

GH at 2 mg/kg/day was also associated with a higher final body weight than untreated WD/CCl_4_ mice. Importantly, this difference was not accompanied by greater liver weight, visceral adipose tissue mass, or liver-to-body or adipose tissue-to-body weight ratios. Thus, the increase in body weight was not associated with hepatomegaly or increased visceral adiposity. Although food intake, energy expenditure, body composition, and lean mass were not measured, the anthropometric findings are consistent with a systemic response to GH that occurred without detectable expansion of the major pathological tissue compartments assessed in this study. Further work will be needed to define whether this effect reflects changes in lean mass, hydration, or other components of body composition.

All four hormone regimens reduced serum GPT/ALT and GOT/AST levels relative to untreated WD/CCl_4_ mice. These biochemical improvements were accompanied by lower NAS scores across all treatment groups. We previously showed that in an earlier-stage MASLD model, GH and IGF-1 supplementation can reverse hepatic steatosis and normalize transaminases [25]. A clinical meta-analysis also reported improvements in hepatic lipid content, visceral adiposity, and inflammatory markers following GH therapy in patients with MASLD [58]. Concordance with our previous findings suggests that the beneficial effects of GH and IGF-1 on liver injury are maintained in this more advanced disease setting, despite the presence of established MASLD-related HCC.

The expression of lipogenesis-related markers was heterogeneous but provided evidence of selective metabolic modulation. IGF-1 at 20 µg/kg/day was associated with lower SREBP-1c mRNA, suggesting a possible reduction in one transcriptional component of lipogenic regulation. This change was not accompanied by lower ACC or FASN mRNA or by a reduction in FASN protein. Because SREBP-1c activity is regulated not only at the transcriptional level but also through proteolytic processing and nuclear translocation [59,60,61], the observed decrease in mRNA should not be interpreted as definitive evidence of reduced hepatic de novo lipogenesis. GH at 0.5 mg/kg/day increased ACC mRNA without altering SREBP-1c or FASN mRNA or FASN protein abundance. This change was accompanied by higher hepatic GHR mRNA expression. A previous study, have shown that hepatocyte GHR signaling regulates de novo lipogenesis through direct and indirect mechanisms that depend on the metabolic and nutritional context [62]. However, the parallel increases in ACC and GHR mRNA do not establish a causal relationship, as receptor activation, ACC protein abundance, and lipogenic activity were not assessed. Although ACC-dependent lipogenesis has been implicated in HCC development [63,64], the increase in ACC mRNA was not accompanied by a detectable increase in nodule burden in our model. In contrast, GH at 2 mg/kg/day increased FASN protein abundance despite unchanged FASN mRNA. A similar discrepancy between transcript and protein levels has been reported in HCC tissue, with FASN protein levels increased in tumoral tissue despite unchanged FASN mRNA levels [65]. Experimental studies have also identified mechanisms regulating FASN translation and protein stability [66,67,68], although these processes were not evaluated in our study. Interestingly, the increase in FASN protein occurred alongside reduced hepatic steatosis and lobular inflammation, while GH at 0.5 mg/kg/day reduced ballooning and lobular inflammation. These findings emphasize that GH and IGF-1 influenced specific components of hepatic lipid metabolism rather than producing a uniform lipogenic response.

The fibrosis-related findings showed differences between the two doses evaluated at the transcript level. IGF-1 at 60 µg/kg/day was associated with lower COL1A1, CTGF, and TIMP-1 mRNA, whereas GH at 2 mg/kg/day was associated with lower CTGF, TIMP-1, and MMP2 mRNA. IGF-1 at 20 µg/kg/day also reduced CTGF expression. These observations are compatible with previous evidence that IGF-1 can influence hepatic stellate-cell activity, senescence, and extracellular matrix turnover [69,70,71]. GH may likewise affect hepatocyte repair and hepatic transcriptional programs involved in the response to injury [31,72,73]. The transcriptional changes were accompanied by a significant reduction in hepatic collagen-positive area, as assessed by Masson’s trichrome staining, although collagen type I protein abundance did not differ significantly between treated and untreated WD/CCl_4_ mice. This discrepancy may reflect differences between the endpoints, as hepatic fibrosis involves the accumulation of different collagen types and remodeling of the extracellular matrix [74], as Masson’s trichrome staining evaluates collagen deposition and distribution within the tissue, whereas Western blotting measures the relative abundance of one type of collagen protein in liver homogenates. Furthermore, histological fibrosis staging also considers the distribution and architectural pattern of collagen deposition, ranging from perisinusoidal or portal/periportal fibrosis in early stage (F1-F2) to bridging fibrosis (F3) and cirrhosis (F4) [75]. In this context, the lower collagen-positive area observed in IGF-1- and GH- treated mice was accompanied by a histological pattern consistent with less advanced fibrosis (F1c-F2), compared with bridging fibrosis (F3) in untreated WD/CCl_4_ mice.

The inflammatory response also differed according to treatment and dose. IGF-1 at 60 µg/kg/day reduced MCP-1 mRNA, providing evidence of a potentially favorable effect on one chemokine associated with inflammatory recruitment. GH at 0.5 mg/kg/day increased IL-1β expression, but this change was not accompanied by higher IL-6 or MCP-1 relative to untreated WD/CCl_4_ mice. GH at 2 mg/kg/day did not significantly alter the evaluated inflammation-related transcripts. Despite this heterogeneous molecular response, all hormone-treated groups showed reduced lobular inflammation and serum aminotransferase levels. A previous experimental studies have shown that IL-1β-induced inflammatory signaling alone does not necessarily reproduce the histological features of MASH [76]. Furthermore, IL-1β activity depends on inflammasome-mediated processing, indicating that increased transcript abundance does not necessarily reflect greater mature cytokine activity [77]. Thus, the increase in IL-1β mRNA observed with GH at 0.5 mg/kg/day cannot be interpreted as evidence of increased inflammatory activity, particularly given the concurrent reduction in lobular inflammation. However, because we did not assess inflammatory protein levels or downstream signaling, the mechanisms underlying the histological improvement remain unclear.

The reduction in hepatic surface nodule burden with GH at 2 mg/kg/day is the most consequential finding. This group had fewer nodules per mouse at the study endpoint. In contrast, neither dose of IGF-1 nor GH at 0.5 mg/kg/day significantly altered the evaluated nodule outcomes. The fact that GH at 2 mg/kg/day resulted in a favorable lesion phenotype, unlike the 0.5 mg/kg/day dose, highlights the importance of dose for determining the biological effects of GH. IGF-1 modified biochemical and fibrosis-related markers without increasing nodule burden, despite experimental evidence that IGF-1 can support HCC-cell growth under some conditions [78]. Neither IGF-1 dose was associated with an increase in macroscopic hepatic lesion burden in this model. These results also suggest that changes in liver injury or fibrogenic markers are not necessarily accompanied by corresponding changes in macroscopic hepatic lesions. These results are reassuring in that neither IGF-1 dose promoted the macroscopic lesion burden in this model, while also demonstrating that improvements in liver injury or fibrogenic markers do not necessarily predict changes in hepatic lesions.

The nodule findings require two qualifications. First, the lesions were identified macroscopically and were not reported as histopathologically classified HCC. Adenomas, preneoplastic foci, and regenerative nodules can also produce visible hepatic lesions. The endpoint should therefore be described as hepatic surface nodule burden unless histological confirmation is available. Second, tumor status was not assessed in the same animals before hormone administration. The study can support an effect on nodule development during late-stage hepatocarcinogenesis but cannot distinguish prevention of new lesions from reduced growth or regression of lesions already present. Even with these limitations, the lower lesion burden associated with GH at 2 mg/kg/day provides a valuable basis for future studies using serial imaging and histopathological classification.

The ERK results provide a preliminary molecular correlate of the lower hepatic surface nodule burden observed with GH at 2 mg/kg/day. In this exploratory analysis (*n* = 3 per group), p-ERK abundance was lower in nodular regions from GH-treated mice, without difference in non-nodular regions. Total ERK abundance was unchanged, while the p-ERK/ERK ratio was lower in both nodular and non-nodular regions from GH-treated mice. Representative immunohistochemical images suggested less nuclear p-ERK staining in GH-treated liver than in untreated WD/CCl_4_ liver. These findings may be relevant in the context of previous studies associating RAF–MEK–ERK pathway activation with HCC progression [79,80] and ERK phosphorylation with the response to sorafenib-based treatment in HCC [81,82].

However, these findings should be interpreted considering the limited number of samples (*n* = 3 per group) and differences in tissue composition, as whole-liver samples from untreated WD/CCl_4_ mice were compared with nodular and non-nodular regions from GH-treated mice. The present data therefore does not establish that altered ERK signaling mediates the lower hepatic nodule burden. Further studies using larger cohorts are needed to determine whether changes in ERK phosphorylation contribute to the lower hepatic nodule burden associated with GH treatment.

The transcriptional profile of the somatotropic axis provides additional evidence of a dose-specific hepatic response. GH at 2 mg/kg/day did not increase GHR, IGF-1, or IGF-1R mRNA relative to untreated WD/CCl_4_ mice. In contrast, GH at 0.5 mg/kg/day increased GHR mRNA and showed higher IGF-1R expression than the 2 mg/kg/day group. The dose associated with lower nodule burden was therefore not accompanied by transcriptional upregulation of the measured somatotropic-axis components. This finding does not demonstrate receptor desensitization or GH resistance, because receptor protein abundance, circulating GH and IGF-1, and canonical downstream signaling were not assessed. It does, however, show that the two GH doses produced distinct transcriptional profiles and that the favorable lesion response at the higher dose was not simply explained by increased expression of these measured genes.

The relationship between GH signaling and cancer is strongly dependent on biological context. Chronic GH excess and sustained activation of GH/IGF signaling have been associated with tumor-promoting effects, whereas long-term replacement studies have not consistently demonstrated a general increase in cancer incidence [83]. Conversely, congenital disruption of GH or IGF-1 signaling is associated with lower cancer incidence in humans and increased longevity and cancer resistance in experimental models [84,85]. The present findings do not resolve this broader question. They show that, within a specific late-stage WD/CCl_4_ model, continuous GH administration at 2 mg/kg/day was not associated with greater hepatic nodule burden and instead coincided with fewer nodules, decreased to hepatic collagen, lower ERK phosphorylation, and improvement in serum aminotransferases. This result supports a context- and dose-specific interpretation of GH action and indicates that GH is not uniformly pro-tumorigenic in this experimental setting.

The mode of hormone delivery is another relevant consideration. GH was administered continuously through osmotic pumps, whereas endogenous GH secretion in male mice is pulsatile. The temporal pattern of GH exposure influences hepatic GHR–JAK2–STAT5 signaling and sex-dependent transcriptional programs [86,87,88]. Continuous exposure may therefore produce biological effects that differ from those of intermittent administration or physiological replacement. In addition, the absence of hormone-treated chow controls prevents separation of disease-specific effects from the consequences of prolonged GH or IGF-1 exposure to otherwise healthy animals. Previous studies indicate that exogenous GH can affect liver growth, hepatocyte size, proliferation, and hepatic signaling in healthy mice, with effects depending on the dose and administration schedule [86,87,88]. Evidence regarding prolonged IGF-1 administration is more limited [89,90]. These considerations highlight the need to evaluate whether the favorable effects observed with GH at 2 mg/kg/day are maintained under alternative dosing schedules and in additional experimental contexts.

Several additional limitations should be considered. The study used a single chemically accelerated model and included only male mice. The absence of CCl_4_-only experimental groups also prevented us from distinguishing the relative contributions of metabolic and chemically induced liver injury. However, the study was designed to evaluate the effects of GH and IGF-1 within the combined WD/CCl_4_ model. Therefore, our findings should be interpreted as treatment effects within this specific experimental context and do not establish whether the observed responses are driven predominantly by the metabolic, fibrogenic, or combined components of the model. Sample sizes differed among assays, and numerous molecular outcomes were evaluated without a clearly defined primary endpoint. Hormone exposure and pharmacodynamic target engagement were not measured. The study also lacked histopathological classification of the surface nodules, and comprehensive safety assessments. These limitations affect the strength of the conclusions, but they also define clear priorities for future work. In particular, the combination of lesion quantification, reduced hepatic collagen, ERK pathway analysis, aminotransferase improvement, and fibrosis-related transcriptional changes provide a useful framework for more definitive studies.

Within these constraints, the results identify GH at 2 mg/kg/day as the only intervention associated with a lower hepatic surface nodule burden. This phenotype was accompanied by reduced hepatic steatosis and lobular inflammation, lower serum aminotransferases, reduction collagen hepatic, changes in selected fibrosis-related transcripts, and reduced ERK phosphorylation, despite an increase in FASN protein abundance. Together, these findings suggest that GH at 2 mg/kg/day was associated with a distinct hepatic phenotype characterized by reduced macroscopic lesion burden and modulation of pathways relevant to hepatocarcinogenesis. IGF-1 improved selected biochemical and fibrosis-related endpoints without altering nodule burden and did not increase lesion formation. The findings therefore support a model in which GH and IGF-1 produce distinct hepatic responses according to the administered dose during late-stage WD/CCl_4_-induced hepatocarcinogenesis.

Confirmation will require histopathological classification of the lesions, pharmacodynamic measurements, and replication in additional models and both sexes. The present results are hypothesis-generating, but they provide a positive foundation for investigating how carefully defined GH dosing may influence liver injury, fibrogenic signaling, and lesion development in MASLD-related HCC.

## 4. Materials and Methods

Animals, diet, and experimental design: Male C57BL/6J mice were used for the experimental studies from the Animal Facility of the Department of Gastroenterology, Pontificia Universidad Católica de Chile, following the animal use and care protocols reviewed and approved by the committee of animal care and well-being of the university’s School of Medicine (ID: 18080912, 22 April 2019). Mice were maintained on a 12:12 h light–dark cycle at 23 °C and housed in cages with a maximum of three mice per cage; cage order and animal location were managed according to institutional protocols to minimize handling and confounders. We provided water and a standard chow diet (Prolab 3000, Purina, PMI Feeds Inc., St. Louis, MO, USA) ad libitum before initiating the study. At nine-week-old, mice were randomly assigned to experimental groups. The Chow group (control) received a standard chow diet and tap water throughout the experiment. To induce MASLD, experimental mice were fed a western diet (WD; Envigo Teklad diets, TD.120528, Madison, WI, USA) containing 21.1% fat, 41% sucrose, and 1.25% cholesterol and given high-sugar drinking water containing fructose (23.1 g/L) and glucose (18.9 g/L). The diet was administered for 24 weeks. Hepatocarcinogenesis was induced simultaneously with diet initiation through intraperitoneal injections of carbon tetrachloride (CCl_4_) in corn oil (vehicle) at a dose of 0.2 µL/g of body weight (equivalent to approximately 0.32 mg/g) once per week, continuing throughout the 24-week experimental period [57,91]. The combined WD/CCl_4_ protocol was used as an established model of advanced MASLD-related liver injury and HCC. The study evaluated the effects of GH and IGF-1 within this established disease context rather than dissecting the independent contributions of Western diet and CCl_4_.

**Figure 6:**
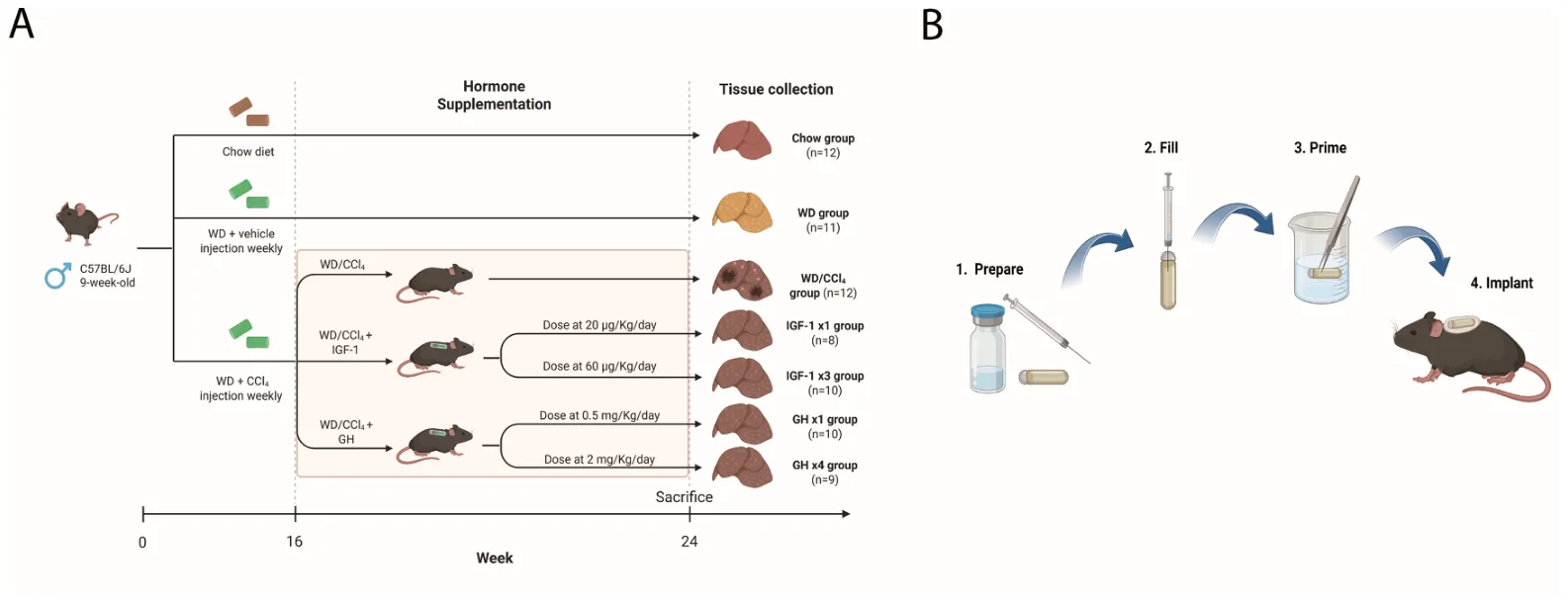
Experimental design and establishment of a MASLD-related HCC mouse model. (**A**) Schematic representation of the experimental approach for each of the study groups. (**B**) Implantation of osmotic pumps. Mice were fed a chow or western diet (WD) for 24 weeks. WD-fed mice received weekly CCl_4_ injections. Osmotic pumps were implanted subcutaneously in C57BL/6J mice after 16 weeks of a WD/CCl_4_ diet for continuous subcutaneous infusion of two doses of insulin-like growth factor type 1 (IGF-1) (IGF-1 x1: 20 µg/kg/day; IGF-1 x3: 60 µg/kg/day) or two doses of growth hormone (GH) (GH x1: 0.5 mg/kg/day; GH x 4: 2 mg/kg/day), during the last 8 weeks.

After 16 weeks of WD and CCl_4_ exposures, mice were randomly allocated to treatment groups and received hormonal supplementation for the remaining 8 weeks. Experimental groups included: Chow group (*n* = 12), WD group (*n* = 11), WD/CCl_4_ group (*n* = 12), IGF-1 x1 group (20 µg/kg/day, *n* = 8); IGF-1 x3 group (60 µg/kg/day, *n* = 10), GH x1 group (0.5 mg/kg/day, *n* = 10), and GH x4 group (2 mg/kg/day, *n* = 9). Hormones were delivered continuously via subcutaneous osmotic pumps until study completion (Figure 6A,B). We euthanized the mice under anesthesia by exsanguination, then collected serum, adipose, and liver tissues and stored them at −80 °C for subsequent analyses.

Hormone treatment and administration via osmotic pumps: We selected GH and IGF-1 doses based on previous preclinical studies demonstrating the biological activity of somatotropic supplementation in metabolic and chronic liver disease models. In a previous study using high-fat diet-fed mice, GH doses ranging from 0.005 to 5 mg/kg/day were evaluated, with 0.5 and 5 mg/kg/day producing measurable hepatic and metabolic effects [92]. Accordingly, we selected a GH dose of 0.5 mg/kg/day, which had shown measurable hepatic effects in the previous dose-ranging study. In our previous MASLD model, GH administered at 9 mg/kg/day also produced hepatoprotective effects; however, this exposure was accompanied by hepatomegaly and hyperinsulinemia [25]. Because the present study was conducted in a more advanced disease context characterized by advanced fibrosis and hepatocarcinogenesis, we deliberately selected a GH dose of 2 mg/kg/day, representing a four-fold increase over 0.5 mg/kg/day, remaining below the 5–9 mg/kg/day range previously evaluated in metabolic liver disease models. This selection allowed us to compare two GH doses and explore potential dose-related differences. For IGF-1, 20 µg/kg/day has been used in cases of CCl_4_-induced liver injury and cirrhosis, with improvements in biochemical, histological, and molecular indicators of liver damage [93]. Additionally, this dosage showed significant hepatic effects in our previous MASLD model [25]. More recently, the same IGF-1 dose was used in mice fed a choline-deficient high-fat diet to investigate restoration of dysregulated somatotropic signaling [94]. Based on these findings, we selected an IGF-1 dose of 20 µg/kg/day and increased it three-fold to 60 µg/kg/day. As with GH, the selection of two doses allowed us to explore potential dose-related differences in IGF-1 response. For clarity, we refer to GH at 0.5 mg/kg/day as GH x1 and GH at 2 mg/kg/day as GH x4. We refer to IGF-1 at 20 µg/kg/day as IGF-1 x1, and 60 µg/kg/day as IGF-1 x3.

We achieved continuous delivery of GH or IGF-1 using subcutaneous osmotic pumps (ALZET^®^ Osmotic Pumps, Cupertino, CA, USA) at a continuous flow rate of 0.5 µL/h, according to the manufacturer’s specifications. We dissolved hormones in sterile physiological saline before loading the pumps. We implanted pumps in week 16 and replaced them twice during treatment to ensure continuous hormone delivery over the 8-week supplementation period (Figure 6A,B). For implantation, mice were anesthetized with ketamine (100 mg/kg) and xylazine (10 mg/kg). We shaved the interscapular region and disinfected it with 70% ethanol, followed by povidone–iodine. Ketoprofen (2–5 mg/kg, subcutaneous) was administered prior to surgery for perioperative analgesia. A longitudinal incision was performed in the interscapular region, and a subcutaneous pocket was created by blunt dissection. The osmotic pump was inserted subcutaneously with the delivery portal oriented caudally, avoiding placement directly beneath the incision. The incision was closed with sterile 5–0 silk sutures, and warm sterile saline was applied locally to reduce infection risk. Animals were monitored daily for seven days after surgery to assess wound healing and postoperative recovery.

Macroscopic hepatic nodule assessment: At euthanasia, livers were excised and rinsed in phosphate-buffered saline prior to macroscopic examination. Investigators blinded to treatment groups counted all visible hepatic surface nodules. Nodule diameter was measured using a millimeter ruler immediately after liver collection. Nodules were subsequently categorized by size (≤2 mm or >2 mm) for quantitative analysis.

Biochemical determinations: Serum levels of glutamate-oxaloacetate transaminase (GOT) and glutamate-pyruvate transaminase (GPT) were quantified using commercially available kits from Human Diagnostics Worldwide (Wiesbaden, Germany): GOT (ASAT), IFCC mod. LiquiUV (#12011) and GPT (ALAT) IFCC mod. LiquiUV (#12012). The assay was conducted according to the manufacturer’s instructions, and the absorbance at 340 nm was measured using a double-beam spectrophotometer (UV-150-02; Shimadzu, Tokyo, Japan).

Histological analysis: Liver tissue samples were fixed in 10% formalin, embedded in paraffin, and cut into 5 µm sections. Sections were stained with hematoxylin and eosin (H&E) to evaluate liver histopathology and with Masson’s trichrome to assess collagen deposition and fibrosis. Histological evaluation was performed by two-pathologist blinded to experimental groups. The full spectrum of MASLD features was scored using the system developed by the NASH Clinical Research Network (NASH CRN) [95]. Ten microscopic fields were evaluated per mouse, and each field was scored individually for steatosis (0–3), hepatocyte ballooning (0–2), and lobular inflammation (0–3). The nonalcoholic activity score (NAS) was calculated as the sum of the three components for each field. Images were acquired at 20X magnification with a 50 µm scale bar. The mean NAS across the 10 fields was then calculated for each mouse and used for graphical representation and statistical analysis. To illustrate the histological variability within each experimental group, three microscopic fields per group are shown in Supplementary Figure S5. Fibrosis was staged separately from F0 to F4, assessing the characteristic perisinusoidal zone, portal fibrosis, bridging fibrosis, and cirrhosis. Hepatic collagen-positive area was quantified in four microscopic fields per mouse using Fiji (ImageJ) software (version 1.54p; National Institutes of Health, Bethesda, MD, USA). Images were acquired at 20x magnification with a 50 µm scale bar. Collagen-positive areas were identified by applying a threshold (minimum: 76; maximum 144). The percentage of collagen-positive areas was calculated for each field, and the four values were averaged to obtain a single value per mouse. Group data were then used for graphical representation and statistical analysis.

Immunohistochemistry: In paraffin-embedded tissue sections, antigen retrieval was performed in 0.01 mol/L sodium citrate buffer (pH 6.0) for 20 min. Endogenous peroxidase activity was blocked by incubation with 3% hydrogen peroxide (H_2_O_2_) in methanol for 15 min at room temperature. Tissue sections were incubated for 1 h at room temperature with anti-phospho-ERK 1/2 (Thr202/Tyr 204; #4370, Cell Signaling Technology, Danvers, MA, USA; 1:100) antibody. After washing, tissue sections were incubated with ready-to-use horseradish peroxidase (HRP)-conjugated secondary antibody using the HRP detection kit (#DS-6009-E, Diagnostic BioSystems Inc., Pleasanton, CA, USA) for 15 min at room temperature. Immunoreactivity was visualized using 3,3-Diaminobenzidine (DAB; Dako, Agilent Technologies, Glostrup, Denmark) as chromogen in the presence of H_2_O_2_. Nuclei were counterstained with Harris hematoxylin. Slides were dehydrated through graded alcohols, cleared in xylene, and mounted with coverslips using ProLong™ Diamond Antifade Mountant (#P36961, Molecular Probes, Thermo Fisher Scientific, Waltham, MA, USA).

Western blotting: Liver tissue was homogenized in RIPA lysis buffer [50 mmol/L Tris (pH 8), 1% Triton X-100, 0.1% SDS, 150 mmol/L NaCl, 5 mmol/L EDTA, and 0.5% sodium deoxycholate] supplemented with 1X protease/phosphatase inhibitor cocktail (#5872, Cell Signaling Technology). Lysates were centrifuged at 14,000 rpm for 15 min at 4 °C, and protein concentration was determined using the Pierce^TM^ BCA Protein Assay Kit (#23227, Thermo Fisher Scientific, Waltham, MA, USA). Protein extract (20–30 µg per sample) was separated by electrophoresis in a 10–12% SDS-PAGE and then transferred onto 0.45 µm PVDF membranes. Then, PVDF membranes were blocked with 5% bovine serum albumin (BSA) in Tris-buffered saline containing 0.1% Tween-20 (TBST) for 1 h at room temperature. The blot was incubated overnight at 4 °C with primary antibodies against phospho-ERK 1/2 (Thr202/Tyr204; #4370, Cell Signaling Technology; 1:1000), total ERK 1/2 (#9102, Cell Signaling Technology; 1:1000), FASN (ab22759, Abcam, Cambridge, UK; 1:1000), collagen Type I (ab34710, Abcam, 1:500), and vinculin (sc-73614, Santa Cruz Biotechnology, Dallas, TX, USA; 1:3000). After washing, membranes were incubated with appropriate HRP-conjugated secondary antibodies (Invitrogen, Thermo Fisher Scientific, Waltham, MA, USA) for 1–2 h at room temperature. We detected the reaction using the SuperSignal^TM^ West Dura Extended Duration Substrate (#34075, Thermo Scientific) on a G Box system (Syngene, Synoptics Ltd., Cambridge, UK). We performed densitometric analysis using Image Lab software (version 6.1, build 43; Bio-Rad Laboratories, Hercules, CA, USA). Phosphorylated ERK, total ERK, FASN, and collagen type I were quantified separately and normalized to the loading control vinculin. The p-ERK/ERK ratio was subsequently calculated using the vinculin-normalized values.

RNA extraction, reverse transcription, and quantitative real-time PCR: Total RNA from liver tissue was isolated using an SV Total RNA Isolation System (#Z3100, Promega, Madison, WI, USA) according to the manufacturer’s instructions. The RNA purity was assessed by measuring the 260/280 absorbance ratio using a NanoDrop ND-1000 spectrophotometer (NanoDrop Technologies, Wilmington, DE, USA). Subsequently, cDNA was synthesized from 1 µg of total RNA using the ImProm-II^TM^ Reverse Transcription System (#A3800, Promega) in the Maxy Gene^TM^ II Thermal Cycler (THERM-1001; Axygen, Corning Incorporated, Corning, NY, USA). The resulting cDNA was serially diluted 1:10 to 1:50, and quantitative real-time PCR (qRT-PCR) was performed with Power-Up™ SYBR Green Master Mix (#A25742, Applied Biosystems, Thermo Fisher, Waltham, MA, USA) in the QuantStudio 3 Real-Time PCR System (Applied Biosystems). The relative expression of each gene was calculated using the comparative 2^−ΔΔ*Ct*^ method after normalization to three endogenous controls (RPLP0, B2M, and HPRT-1). Data were normalized to a value of 1 in the chow group. Primer sequences for qRT-PCR are detailed in Supplementary Table S2.

Statistical analysis: Data are presented as mean ± SEM. Statistical analyses were performed using GraphPad Prism version 8.0 (GraphPad software, San Diego, CA, USA). The number of animals per group was selected based on previous preclinical studies evaluating the effects of GH or IGF-1 in models of liver disease [72,96,97,98], as well as our previous studies using a MASLD murine model [24,25]. Normality of the data distribution was assessed using the Shapiro–Wilk test. For comparisons between two groups, an unpaired Student’s *t*-test was applied to normally distributed data, whereas the Mann–Whitney U test was used for non-normally distributed data. For comparisons among multiple groups, one-way or two-way ANOVA was performed as appropriate, followed by Tukey’s post hoc test for parametric data or Kruskal–Wallis test with Dunn’s multiple comparison correction for non-parametric data. Categorical variables, including nodule size distribution, were analyzed using the chi-square or Fisher’s exact test; transaminase levels were analyzed using mixed-effects models. A *p*-value < 0.05 was considered statistically significant.

## 5. Conclusions

In this murine model of advanced MASLD-related HCC, late-phase administration of GH and IGF-1 administration was associated with lower serum aminotransferase levels, lobular inflammation, and NAS scores. The effects on steatosis, hepatocyte ballooning, and fibrosis-related transcripts varied according to hormone and dose. The fibrosis-related findings suggest that both GH and IGF-1 may favorably influence hepatic collagen deposition, although the underlying mechanism remains to be established.

GH at 2 mg/kg/day was the only regimen associated with a lower incidence and number of macroscopically detected hepatic surface nodules. This finding was accompanied by a lower hepatic p-ERK/ERK ratio, although a causal relationship between ERK phosphorylation and nodule burden was not established. Neither GH at 0.5 mg/kg/day nor either dose of IGF-1 significantly altered the evaluated nodule outcomes.

These findings suggest a dose-specific association between GH administration and hepatic surface nodule burden during late-stage WD/CCl_4_-induced hepatocarcinogenesis. However, the absence of histopathological classification of the nodules and assessment of lesion status before treatment prevents conclusions regarding HCC regression or reduced HCC burden. The use of a single murine model, the inclusion of only male mice, and the lack of pharmacodynamic measurements further limit generalization. Histopathological confirmation, longitudinal assessment of lesion development, and validation in additional experimental models are required to determine whether this association is reproducible and to clarify the mechanisms involved.

## Data Availability

The data presented in this study are available from the corresponding authors upon reasonable request.

## Supplementary Materials

The following supporting information can be downloaded at https://www.mdpi.com/article/10.3390/ijms27219985/s1.

## Abbreviations

MASLD	Metabolic dysfunction-associated steatotic liver disease
HCC	Hepatocellular carcinoma
SAD	Somatotropic axis dysfunction
WD	Western diet
CCl_4_	Carbon tetrachloride
GH	Growth hormone
IGF-1	Insulin-like growth factor 1
GPT	Glutamate-pyruvate transaminase
GOT	Glutamate-oxaloacetate transaminase
H&E	Hematoxylin and eosin
