# Supplementary material for: Growth Hormone Reduces Liver Tumor Development in a MASLD-Related HCC Murine Model
Source: Int J Mol Sci. 2026 Nov 7;27(21):9985. doi: 10.3390/ijms27219985 (PMC13764173; doi:10.3390/ijms27219985)
Supplement: Supplementary file 1 [file ijms-27-09985-s001.zip › ijms-4428156-supplementary.pdf]

Supplementary Materials:

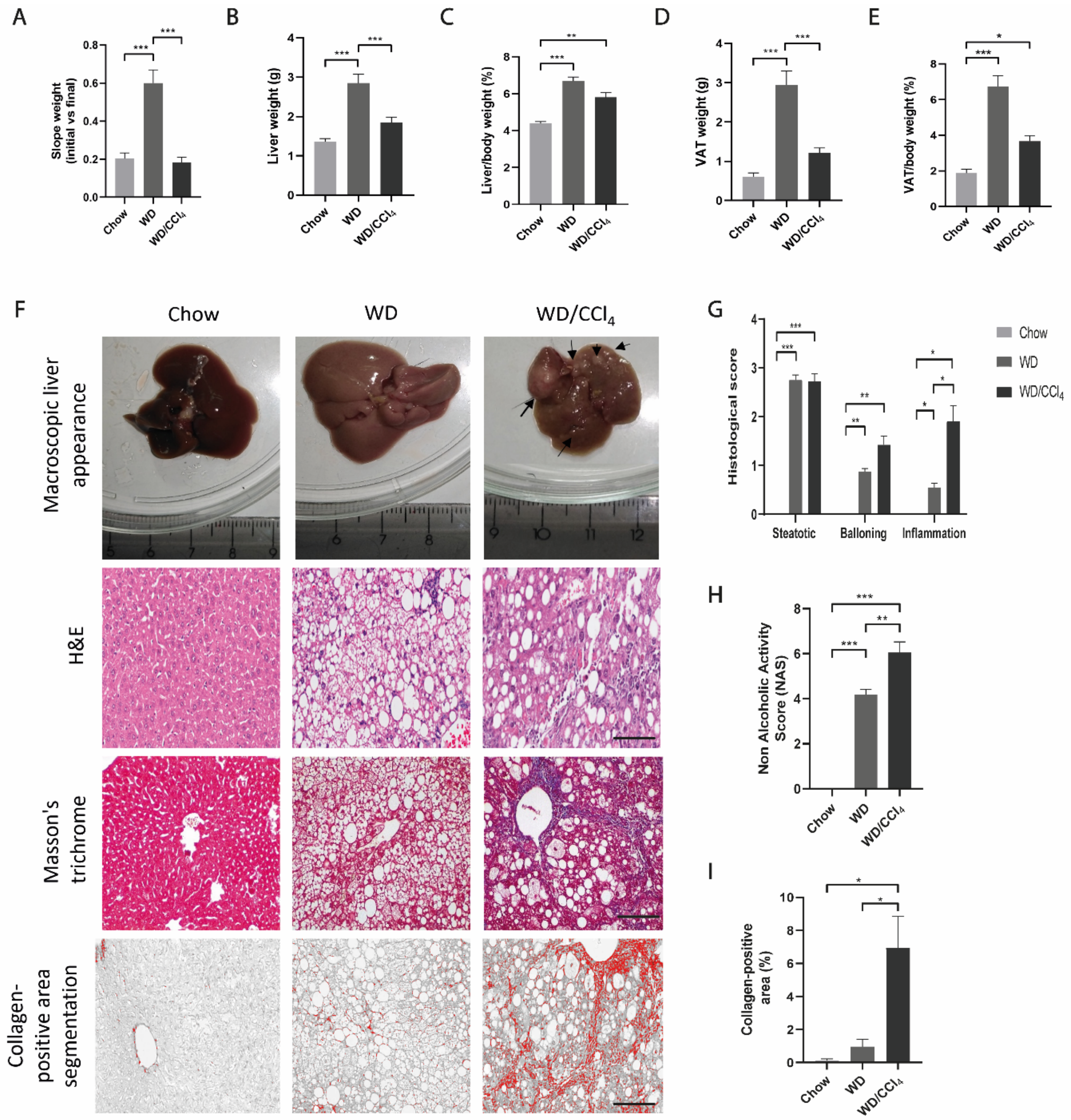

Supplementary Figure S1. Successful Induction of MASLD- related HCC Prior to Therapeutic Intervention. (**A**) Slope graph showing body weight at the beginning and at the end of the experimental protocol (*** $p < 0.001$, one-way ANOVA). (**B**) Liver weight (g) (*** $p < 0.001$; one-way ANOVA). (**C**) Liver-to-body weight ratio (%) (** $p < 0.01$; *** $p < 0.001$, Kruskal–Wallis). (**D**) Visceral adipose tissue (VAT) weight (g) (*** $p < 0.001$, one-way ANOVA). (**E**) VAT-to-body ratio (%) (* $p < 0.05$; *** $p < 0.001$, Kruskal–Wallis) (Chow, $n = 12$; WD, $n = 11$; WD/CCl_4, $n = 12$) (**F**) Representative macroscopic appearance of liver (upper panels), hematoxylin and eosin (H&E)-stained liver sections (second panels), Masson's trichrome staining (third panels), and collagen-positive area segmentation using ImageJ software (lower panels) from mice fed a chow diet (Chow), Western diet (WD), or Western diet plus CCl_4 treatment (WD/CCl_4). Collagen fibers stain blue, cytoplasm and muscle fibers stain red, and nuclei appear dark. Black arrows indicate tumoral nodules. Scale bar 50 µm. (**G**) Histological score (Chow, $n = 5$; WD, $n = 4$; WD/CCl_4, $n = 4$) (* $p < 0.05$; ** $p < 0.01$, *** $p < 0.001$, two-way ANOVA). (**H**) nonalcoholic fatty liver disease

activity score (NAS) (Chow, *n* = 5; WD, *n* = 4; WD/CCl_4, *n* = 4; * $p < 0.05$, ** $p < 0.01$, *** $p < 0.001$, one-way ANOVA). (**I**) Quantification of collagen-positive area (%) in Masson´s trichrome-stained liver section. (Chow, *n* = 2; WD, *n* = 3; WD/CCl_4, *n* = 3; * $p < 0.05$, one-way ANOVA). Data are presented as mean ± SEM.

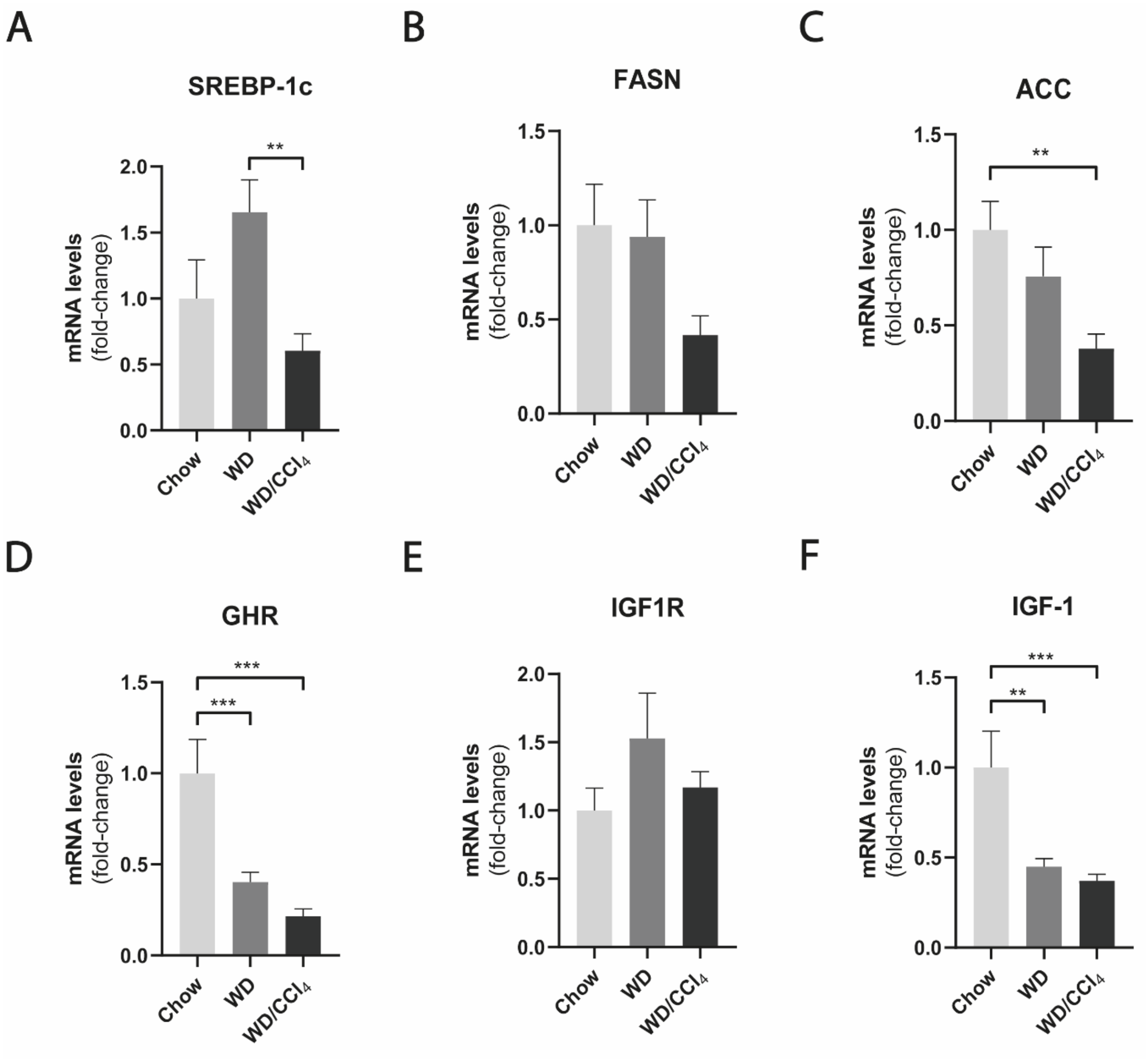

Supplementary Figure S2. Effects of lipogenic and somatotropic axis-related genes in MASLD-induced HCC. (**A-C**) Hepatic mRNA expression levels of sterol regulatory element-binding protein 1 (SREBP-1c), Acetyl-CoA carboxylase (ACC), and fatty acid synthase (FASN) (Chow, *n* = 5; WD, *n* = 7; WD/CCl_4, *n* = 8; ** $p < 0.01$, one-way ANOVA). (**D-F**) mRNA expression level of insulin-like Growth factor 1 (IGF-1), insulin-like growth factor 1 receptor (IGF-1R), and growth hormone receptor (GHR) (Chow, *n* = 5; WD, *n* = 7; WD/CCl_4, *n* = 8; * $p < 0.05$; ** $p < 0.01$; *** $p < 0.001$, one-way ANOVA or Kruskal–Wallis). Data are presented as mean ± SEM.

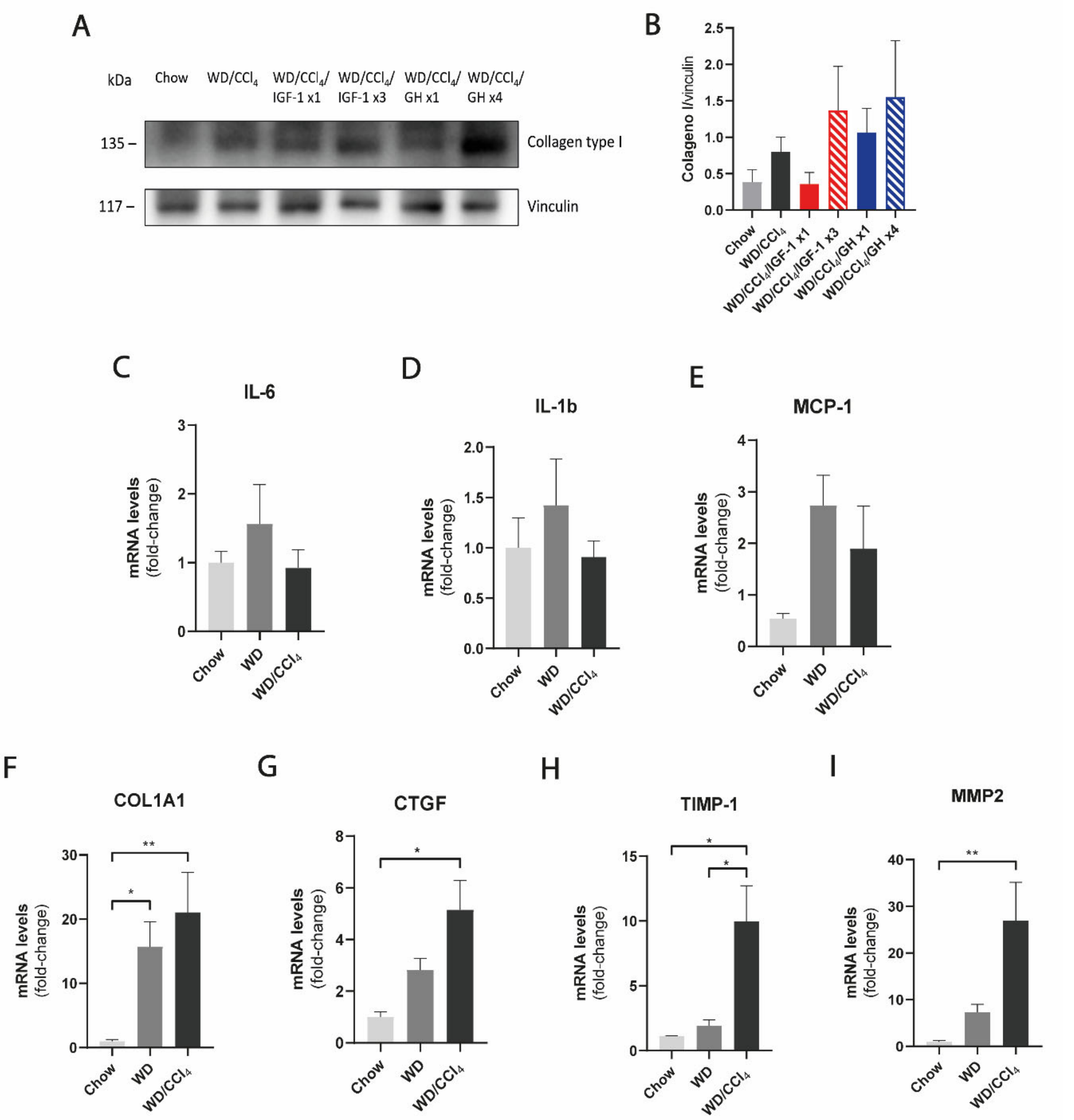

Supplementary Figure S3. CCl_4-induced chemical carcinogenesis promotes a fibrotic transcriptional profile in liver injury. (**A**) Western blot analyses of Collagen type I protein levels in hepatic tissue. Vinculin was used as a loading control. Numbers on the left side of the panel indicate molecular weight (kDa). (**B**) Densitometric quantification of Collagen type I protein levels normalized to control loading vinculin (Chow, $n = 5$; WD/CCl_4, $n = 5$; WD/CCl_4/IGF-1 x1, $n = 7$; WD/CCl_4/IGF-1 x3, $n = 6$; WD/CCl_4/GH x1, $n = 8$; WD/CCl_4/GH x4, $n = 6$). (**C-E**) qPCR was performed to assess the mRNA level of interleukin 6 (IL-6), interleukin 1 beta (IL-1β), and monocyte chemoattractant protein-1 (MCP-1), which are inflammation-related genes (Chow, $n = 5$; WD, $n = 7$; WD/CCl_4, $n = 8$; not significant, one-way ANOVA or Kruskal–Wallis). (**F-I**) qPCR was performed to assess the mRNA levels of α-1 type I collagen (COL1A1), connective tissue growth factor (CTGF), tissue inhibitor of metalloproteinase-1 (TIMP-1), and matrix metalloproteinase 2 (MMP2), which are fibrosis-related genes (Chow, $n = 5$; WD, $n = 7$; WD/CCl_4, $n = 8$; * $p < 0.05$; ** $p < 0.01$, one-way ANOVA or Kruskal–Wallis). Data are presented as mean ± SEM.

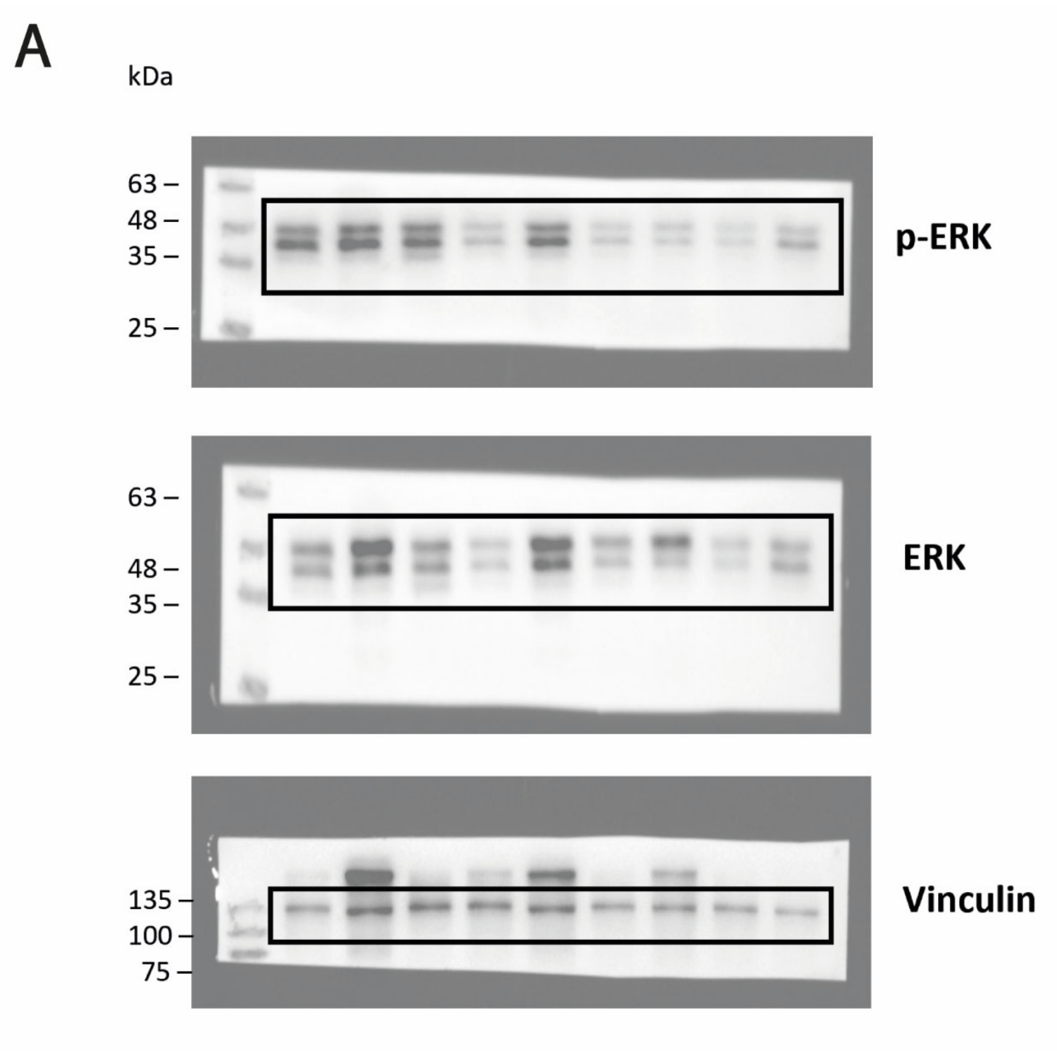

Supplementary Figure S4. (**A**) Original immunoblot corresponding to Fig. 5B.

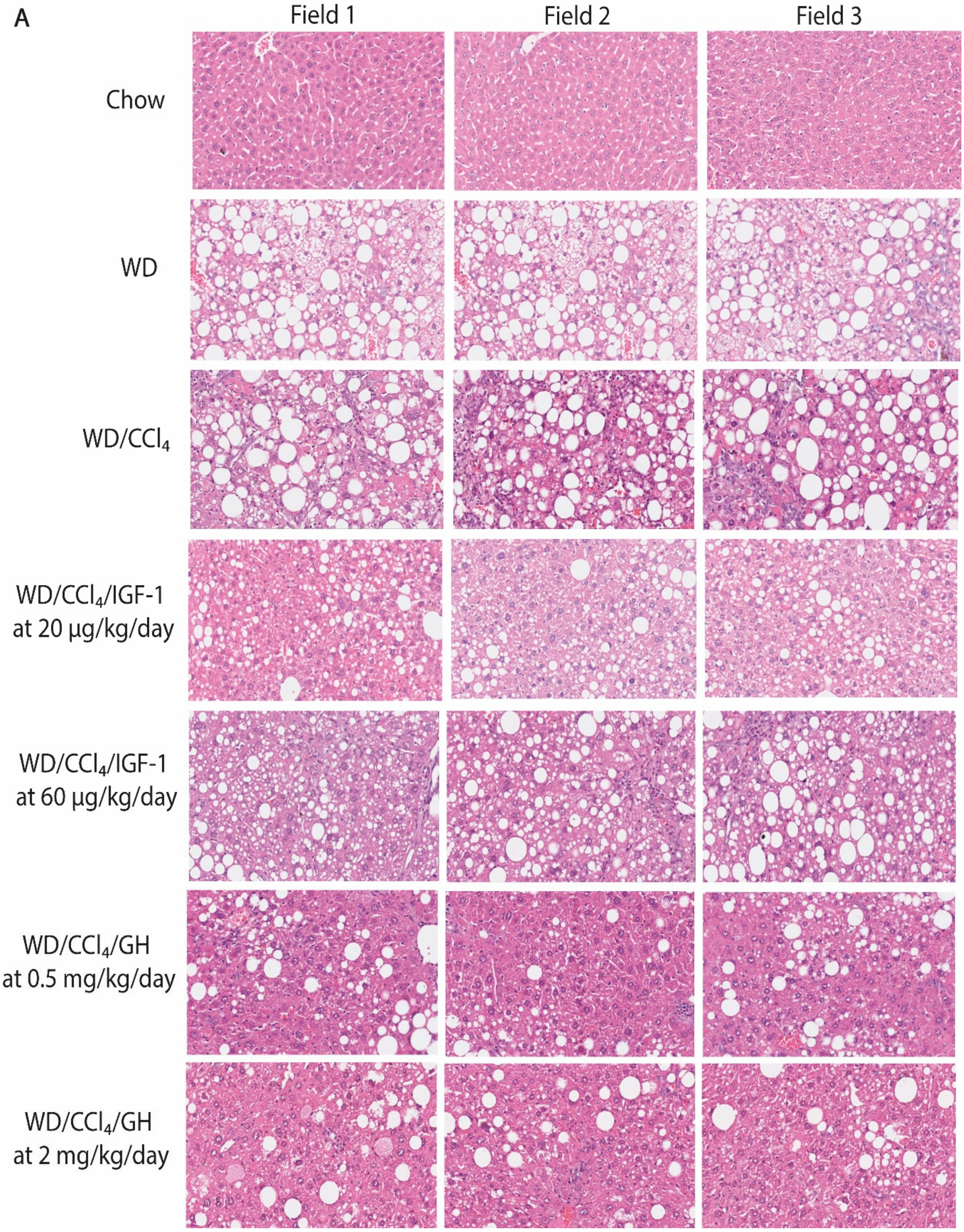

Supplementary Figure S5. Additional representative H&E-stained liver sections from the experimental groups. (**A**) Representative hematoxylin and eosin (H&E)-stained liver sections from mice fed a chow diet (Chow), a Western diet (WD), or a Western diet combined with CCl_4 (WD/CCl_4), with or without IGF-1 (20 or 60 μg/kg/day) or GH (0.5 or 2 mg/kg/day) supplementation. Histological scoring was performed using 10 microscopic fields per mouse, and the mean score was calculated for each animal. Scale bar: 50 μm.

Supplementary Table S1. Descriptive statistics and between-group comparisons of ERK phosphorylation and total ERK protein abundance

A. Descriptive statistics

Parameter	WD/CCl_4 (*n* = 3)	GH x4 non-nodular (*n* = 3)	GH x4 nodular (*n* = 3)
p-ERK/vinculin	3.401 ± 1.600	1.084 ± 1.321	0.392 ± 0.3325
ERK/vinculin	1.969 ± 1.528	2.039 ± 2.793	0.8984 ± 0.6885
p-ERK/ERK	1.073 ± 0.454	0.3338 ± 0.210	0.2137 ± 0.0225

B. Comparison with WD/CCl_4

Parameter	Comparison	Statistical test	Mean difference (95% CI)/ Mean rank diff	adjusted *p* -value
p-ERK/vinculin	WD/CCl_4 vs. GH x4 non-nodular	One-way ANOVA + Dunnet	2.317 (-0.5189 to 5.153)	0.0991
	WD/CCl_4 vs. GH x4 nodular		3.009 (0.1729 to 5.845)	0.04
ERK/vinculin	WD/CCl_4 vs. GH x4 non-nodular	Kruskal–Wallis + Dunn	2	0.7422
	WD/CCl_4 vs. GH x4 nodular		2	0.7422
p-ERK/ERK	WD/CCl_4 vs. GH x4 non-nodular	One-way ANOVA + Dunnet	0.7397 (0.06399 to 1.415)	0.0355
	WD/CCl_4 vs. GH x4 nodular		0.8598 (0.184 to 1.535)	0.0191

Data are presented as mean ± SEM. Comparisons were restricted to WD/CCl_4 versus GH ×4 non-nodular and WD/CCl_4 versus GH ×4 nodular samples. One-way ANOVA followed by Dunnett's multiple comparisons test was used for p-ERK/vinculin and p-ERK/total ERK. The Kruskal–Walli´s test followed by Dunn's multiple comparisons test was used for total ERK/vinculin. Mean differences and their 95% confidence intervals (CI) are reported for parametric comparisons, whereas mean rank differences are reported for Dunn's test. Each group comprised three independent biological samples.

Supplementary Table S2. List of primers used for real-time PCR

Genes	primer sequence (5' ---> 3')		N° Access NCBI
Col1a1	Foward	GCTCCTCTTAGGGGCCACT	NM_007742.3
	Reverse	CCACGTCTCACCATTGGGG	
TIMP-1	Foward	GCAACTCGGACCTGGTCATAA	NM_001044384.1 (transcript Variants)
	Reverse	CGGCCCGTGATGAGAAACT	
MMP2	Foward	CAAGTTCCCCGGCGATGTC	XM_006530751.1 (transcript Variants)
	Reverse	TTCTGGTCAAGGTCACCTGTC	
CTGF	Foward	GGGCCTCTTCTGCGATTTC	NM_010217.2
	Reverse	ATCCAGGCAAGTGCATTGGTA	
MCP-1	Foward	TTAAAAACCTGGATCGGAACCAA	NM_011333.3
	Reverse	GCATTAGCTTCAGATTTACGGGT	
IL-6	Foward	TAGTCCTTCCTACCCCAATTTCC	NM_031168.1
	Reverse	TTGGTCCTTAGCCACTCCTTC	
IL-1β	Foward	ACTGTTCCTGAACTCAACT	XM_006498795.2 NM_008361.3
	Reverse	ATCTTTTGGGGTCCGTCAACT	
SREBP-1c	Foward	GGA GCC ATG GAT TGC ACA TT	XM_006532716.1
	Reverse	GGC CCG GGA AGT CAC TGT	
ACC	Foward	TGACAGACTGATCGCAGAGAAAG	NM_133360.2 (transcript variant)
	Reverse	TGGAGAGCCCCACACACA	
FASN	Foward	GCTGCGGAAACTTCAGGAAAT	NM_007988.3
	Reverse	AGAGACGTGTCACTCCTGGACTT	
IGF-1	Foward	CTGGACCAGAGACCCTTTGC	NM_010512.4 (transcript Variants)
	Reverse	GGACGGGGACTTCTGAGTCTT	
IGF-1R	Foward	GTGGGGGCTCGTGTTTCTC	NM_010513
	Reverse	GATCACCGTGCAGTTTTCCA	
GHR	Foward	AAGCGGACACGAACCCG	NM_010284.3 (transcript Variants)
	Reverse	CTCCAGTTCAGGGGAACGAC	
RPLP0	Foward	CCAGCAGGTGTTTGACAACG	NM_007475.5
	Reverse	TCCAGAAAGCGAGAGTGCAG	
HPRT-1	Foward	GTTGGGCTTACCTCACTGCT	NM_013556.2
	Reverse	TAATCACGACGCTGGGACTG	
B2M	Foward	CATGGCTCGCTCGGTGAC	NM_009735.3
	Reverse	CAGTTCAGTATGTTCGGCTTCC	
